# Risk estimation model for nonalcoholic fatty liver disease in the Japanese using multiple genetic markers

**DOI:** 10.1371/journal.pone.0185490

**Published:** 2018-01-31

**Authors:** Takahisa Kawaguchi, Toshihide Shima, Masayuki Mizuno, Yasuhide Mitsumoto, Atsushi Umemura, Yoshihiro Kanbara, Saiyu Tanaka, Yoshio Sumida, Kohichiro Yasui, Meiko Takahashi, Keitaro Matsuo, Yoshito Itoh, Katsutoshi Tokushige, Etsuko Hashimoto, Kendo Kiyosawa, Masanori Kawaguchi, Hiroyuki Itoh, Hirofumi Uto, Yasuji Komorizono, Ken Shirabe, Shiro Takami, Toshinari Takamura, Miwa Kawanaka, Ryo Yamada, Fumihiko Matsuda, Takeshi Okanoue

**Affiliations:** 1 Center for Genomic Medicine, Kyoto University Graduate School of Medicine, Kyoto, Japan; 2 Department of Gastroenterology and Hepatology, Saiseikai Suita Hospital, Suita, Japan; 3 Department of Surgery, Saiseikai Suita Hospital, Suita, Japan; 4 Center of Hepatology, Nara Municipal Hospital, Nara, Japan; 5 Department of Gastroenterology, Kyoto Prefectural University of Medicine, Kyoto, Japan; 6 Division of Molecular and Clinical Epidemiology, Aichi Cancer Center Research Institute, Aichi, Japan; 7 Department of Internal Medicine and Gastroenterology, Tokyo Women’s Medical University, Tokyo, Japan; 8 Department of Gastroenterology, Nagano Red Cross Hospital, Nagano, Japan; 9 Department of Gastroenterology, Saiseikai Wakayama Hospital, Wakayama, Japan; 10 Department of Gastroenterology, Kure Saiseikai Hospital, Kure, Japan; 11 Digestive and Life-style Related Disease, Kagoshima University Graduate School of Medicine and Dental Science, Kagoshima, Japan; 12 Department of Hepatology, Nanpuh Hospital, Kagoshima, Japan; 13 Department of Surgery and Science, Graduate School of Medical Science, Kyushu University, Fukuoka, Japan; 14 Department of Gastroenterology, Otsu Municipal Hospital, Otsu, Japan; 15 Disease Control and Homeostasis, Kanazawa University Graduate School of Medical Science, Kanazawa, Japan; 16 Center of Liver Disease, Kawasaki Hospital, Kawasaki Medical School, Okayama, Japan; Institute of Medical Research A Lanari-IDIM, University of Buenos Aires-National Council of Scientific and Technological Research (CONICET), ARGENTINA

## Abstract

The genetic factors affecting the natural history of nonalcoholic fatty liver disease (NAFLD), including the development of nonalcoholic steatohepatitis (NASH) and NASH-derived hepatocellular carcinoma (NASH-HCC), are still unknown. In the current study, we sought to identify genetic factors related to the development of NAFLD, NASH, and NASH-HCC, and to establish risk-estimation models for them. For these purposes, 936 histologically proven NAFLD patients were recruited, and genome-wide association (GWA) studies were conducted for 902, including 476 NASH and 58 NASH-HCC patients, against 7,672 general-population controls. Risk estimations for NAFLD and NASH were then performed using the SNPs identified as having significant associations in the GWA studies. We found that rs2896019 in *PNPLA3* [*p* = 2.3x10^-31^, OR (95%CI) = 1.85 (1.67–2.05)], rs1260326 in *GCKR* [*p* = 9.6x10^-10^, OR (95%CI) = 1.38(1.25–1.53)], and rs4808199 in *GATAD2A* [*p* = 2.3x10^-8^, OR (95%CI) = 1.37 (1.23–1.53)] were significantly associated with NAFLD. Notably, the number of risk alleles in *PNPLA3* and *GATAD2A* was much higher in Matteoni type 4 (NASH) patients than in type 1, type 2, and type 3 NAFLD patients. In addition, we newly identified rs17007417 in *DYSF* [*p* = 5.2x10^-7^, OR (95%CI) = 2.74 (1.84–4.06)] as a SNP associated with NASH-HCC. Rs641738 in *TMC4*, which showed association with NAFLD in patients of European descent, was not replicated in our study (*p* = 0.73), although the complicated LD pattern in the region suggests the necessity for further investigation. The genetic variants of *PNPLA3*, *GCKR*, and *GATAD2A* were then used to estimate the risk for NAFLD. The obtained Polygenic Risk Scores showed that the risk for NAFLD increased with the accumulation of risk alleles [AUC (95%CI) = 0.65 (0.63–0.67)]. Conclusions: We demonstrated that NASH is genetically and clinically different from the other NAFLD subgroups. We also established risk-estimation models for NAFLD and NASH using multiple genetic markers. These models can be used to improve the accuracy of NAFLD diagnosis and to guide treatment decisions for patients.

## Introduction

Nonalcoholic fatty liver disease (NAFLD) is frequently associated with metabolic syndrome, a broad range of pathologies including nonalcoholic fatty liver (NAFL), nonalcoholic steatohepatitis (NASH), cirrhosis, and hepatocellular carcinoma (NASH-HCC). NAFLD is classified into four subgroups based on its long-term histological progression. Type 1 (steatosis) and type 2 (steatonecrosis) are classified as NAFL, and type 3 (steatohepatitis) and type 4 (steatohepatitis with fibrosis) as NASH[1]. The prevalence of NAFLD varies widely across the world; for example, it is rare in Asian countries and more common in North America[2]. It was recently reported that the overall NASH prevalence among biopsied NAFLD patients is 59.1%[2].

NAFLD including NASH has a highly varied natural history. In a longitudinal study of 81 NASH and 27 NAFL patients with serial liver biopsies, 45 (42%) patients showed fibrosis progression, while 20 (18%) showed regression after the median follow-up period of 6.6 years[3].

NASH-HCC is considered to be derived mainly from cirrhotic liver, although other factors such as advanced fibrosis and the presence of diabetes mellitus are high risk factors for HCC development[4,5]. Notably, approximately one third of NASH-HCC cases are derived from non-cirrhotic liver[6,7]. These findings indicate that multiple environmental, lifestyle, and genetic factors are involved in its onset and progression. Recent studies demonstrated that, among the pathologic features of NAFLD, only fibrosis independently predicts long-term liver-related mortality [8,9].

The first genome-wide association (GWA) study for NAFLD used NAFLD cases diagnosed by liver fat content, and identified *PNPLA3* as a major genetic determinant for fatty liver and triglyceride content[10] in Hispanic, African American, and European populations. Subsequent studies[6,11–15] showed an association of *PNPLA3* with inflammation, fibrosis, and HCC development. An association of *PNPLA3* with NASH-HCC in European and Japanese populations was demonstrated by genotyping candidate SNPs[6,15,16]. Exome-wide analyses and subsequent replication studies showed an association of *TM6SF2* with NAFLD[17,18]. Rs641738, originally regarded as located in the *MBOAT7* locus but now confirmed to be located in *TMC4*, was initially reported as a susceptibility variant for alcohol-related cirrhosis[19], and was later found to be associated with NAFLD patients of European descent[20]. This variant was not replicated further in any other populations.

We previously reported the results of a GWA study using 529 histologically proven NAFLD cases, that demonstrated that Matteoni type 4 (histologically typical NASH) was both genetically and clinically different from the other three Matteoni types in the Japanese[21]. However, in that study only 29 Matteoni type 3 cases and no NASH-HCC cases were included.

In the present study, we sought to identify genetic factors influencing the development of NAFLD and its progression to NASH-HCC in the Japanese using 902 NAFLD patients. These patients included our previously reported cases, plus 373 new histologically proven cases including 75 type 3 cases and 58 NASH-HCC cases. We also sought to establish risk estimation models for these diseases using multiple genetic markers associated with them.

## Materials and methods

### Study population

A total of 936 NAFLD patients were recruited at 16 hospitals participating in this study consortium. Among them, 888 samples were collected on Honshu Island, while 14 and 34 samples were collected from Kyushu and Shikoku Islands, respectively. Clinical and laboratory data were collected within 14 days before liver biopsy for all of the patients. Inclusion criteria were the same as described in our previous report[21]. Information regarding alcohol usage was obtained from patient surveys. Patients who drank more than 20 g of alcohol per day were excluded. All patients were diagnosed by a hepatopathologist (T.O.) according to Matteoni’s classification[1]. We included 529 cases from our previous study[21], plus 349 new NAFLD and 58 new NASH-HCC patients. The 349 NAFLD cases were classified as 34 type 1, 60 type 2, 84 type 3, and 171 type 4. Non-tumor livers in 58 HCC cases were histologically examined by the same hepatopathologist and diagnosed as type 4 NASH. As a general-population control, 8,364 individual DNA samples of Honshu Island origin consisting of 3,037 collected at Aichi Cancer Center (ACC) including 932 used in our previous study[21], and 5,327 collected for the Nagahama Study[22] were used. In compliance with the Declaration of Helsinki, ethical approval for this study was given by the respective Institutional Review Board and subject written informed consent were obtained for all subjects (Institutional Review Board and Ethics Committee of Kyoto University School of Medicine; Ethical committee of Saiseikai Suita Hospital; Ethics committee of Kyoto Prefectural University of Medicine; Ethical Committee of Aichi Cancer Center; Ethics Committee of Tokyo Women's Medical University; Institutional Review Board of Nagano Red Cross Hospital; Ethical committee of Saiseiken Wakayama Hospital; Ethical committee of Kure Saiseikai Hospital; Ethical committee of Kagoshima University; Ethical committee of Nanpuh Hospital; Kyushu University Institutional Review Board for Clinical Research; Ethical committee of Otsu Municipal Hospital; Medical Ethics Committee of Kanazawa University; Ethical Committee on Kawasaki Medical School and Kawasaki Medical School Hospital; Ethical committee of Nara City Hospital; Ethical Committee of Kochi Medical School, Kochi University; Ethical Committee of Juntendo University; Ethics Committee of Yamagata University School of Medicine; Ethical Committee of the Ikeda Municipal Hospital). Especially, the genome wide association study is approved by IRB at Kyoto University School of Medicine (G1094). All patients were fully informed of the purpose and procedures of this study, and written consent was obtained from each subject.

### SNP genotyping and quality controls

All of the samples were genotyped with SNP arrays provided by Illumina Inc. (San Diego, CA). The genotyping arrays used and numbers of samples are summarized in the S1 Table. After the genotyping samples were subjected to standard quality controls, association analyses were performed for 93,606 SNPs between 844 NAFLD, 58 NASH-HCC, and 7,672 control samples. A detailed description of the quality control processes is available in the S1 Text.

### Statistical analysis

Logistic regression was used for statistical analyses of the GWA studies, comparing 1) all NAFLD patients or 2) NASH-HCC patients with the controls. Population stratification was assessed by the genomic control method[23] and adjusted for 10 principle components (PCs) calculated using a tool for Genome-wide Complex Trait Analysis[24]. Genome-wide significance was set as *p* = 5.3x10^-7^ based on Bonferroni’s correction for multiple testing. Regional genotype imputations were performed with MACH[25] using the 1000 Genomes Project Consortium[26] phase I release version 3 as a template, and SNPs passing an imputation quality threshold of r2>0.5 were used. Linkage disequilibrium (LD) indices were calculated by PLINK[27]. We also conducted a GWA study for the Brunt stage, Brunt grade, and fat-droplet content using ordinal logistic regression. The allele distributions of the genome-wide significant SNPs were compared between the different subgroups of Matteoni types by logistic regression, adjusting for age, sex, BMI, and the 10 PCs.

Polygenic Risk Scores (PRS) were calculated for all NAFLD patients compared with controls. We also calculated the PRS for Matteoni type 4 + NASH-HCC compared with Matteoni type 1 to 3, and for NASH-HCC compared with Matteoni type 4. Using the genome-wide significant SNPs identified in the GWA studies, we generated models by a forward stepwise selection procedure for each comparison, including sex as basic genetic background. PRS were then calculated for each subject using the estimated model, and the study subjects were divided into quintile groups (Q1 to Q5). We compared the lowest quintile group (Q1) with the other groups (Q2 to Q5) using fisher.test in the R package. We also sought to improve the model by adding SNPs previously reported to be associated with NAFLD. We performed additional GWAs for these SNPs, and those with a low p-value (p<1e-4) were included in the models.

## Results

### Characteristics of the study population

The clinical characteristics of the 902 patients and 7,672 control subjects are summarized in Table 1. We compared the distribution of clinical traits between the general-population controls and the Matteoni type 1 subgroup (21 traits), the type 1 and type 2 subgroups (31 traits), the type 2 and type 3 subgroups (31 traits), the type 3 and type 4 subgroups (32 traits), and the type 4 and NASH-HCC subgroups (31 traits). In agreement with our previous study, the results suggested that Matteoni type 4 (histologically typical NASH) was clinically different from the other three Matteoni types. The NASH-HCC and control subjects also showed a clearly different clinical background from the NAFLD patients. A significant difference (*p*<3.5x10^-4^) was observed for 34 of the 146 traits examined, of which 16 were observed between the controls and the type 1 subgroup. There were no significant differences between the type 1 and type 2 or the type 2 and type 3 subgroups, except for the fat-droplet content (*p* = 3.2x10^-4^) between type 1 and type 2. In contrast, six traits, including two biomarkers for liver fibrosis (type IV collagen 7S and hyaluronic acid) were significantly different between the type 3 and type 4 subgroups. These results suggested that Matteoni type 1, type 2, and type 3 belonged to the same subgroup, and Matteoni type 4 formed a distinct subgroup. In the comparison between type 4 and NASH-HCC, 11 clinical traits showed significant differences. All of these 11 markers were associated with the severity of fibrosis, decline of liver function, or a higher age range in the NASH-HCC compared to the type 4 NASH patients.

**Table 1:** Clinical characteristics of the patient populations according to histological classification and the control population enrolled in the study.

Clinical traits	Control	Matteoni classification of NAFLD				NASH-HCC	*p*-value				
		Type 1	Type 2	Type 3	Type 4		Control vs. Type1	Type1 vs. Type2	Type 2vs. Type3	Type3 vs. Type4	Type4 vs. HCC
Number of samples	7672	130	134	104	476	58					
Sex (Male/Female)	3022/4650	79/51	84/50	45/59	192/284	41/17	**1.2x10**^**-6**^**†**	0.85**†**	4.3x10^-3^**†**	0.66**†**	**2.0x10**^**-5**^**†**
Age (year)	52.0±13.4	50.7±15.1	50.9±14.9	52.4±14.1	57.9±14.6	71.5±9.8	0.42	0.98	0.46	**1.1x10**^**-4**^	**8.5x10**^**-11**^
Physical measurement											
BMI	22.4±3.2	26.2±4.3	27.4±4.8	28.1±4.7	27.9±5.1	25.0±2.8	**1.6x10**^**-25**^	0.097	0.19	0.69	8.0x10^-3^
Visceral fat (cm^2^)	-	145.2±65.2	159.7±48.1	137.4±47.0	158.8±55.7	160.5±81.1	-	0.034	0.015	0.020	0.68
Abdominal circumscription (cm)	80.4±9.2	90.1±9.9	93.5±10.2	91.8±9.9	95.1±12.2	94.0±11.0	**1.1x10**^**-11**^	0.091	0.37	0.080	0.87
Biochemical traits											
AST (IU/L)	23.7±10.3	31.5±15.5	37.2±18.7	53.7±38.7	60.0±37.3	52.8±56.0	**8.0x10**^**-14**^	1.4x10^-3^	4.4x10^-4^	3.8x10^-3^	3.4x10^-3^
ALT (IU/L)	21.7±15.9	48.2±31.9	63.6±46.4	82.6±58.6	85.2±66.3	51.7±65.8	**1.2x10**^**-41**^	2.4x10^-3^	9.2x10^-3^	0.81	**1.4x10**^**-8**^
GGT (IU/L)	32.4±45.0	80.5±71.5	71.5±85.9	82.1±74.2	80.2±72.7	108.4±123.7	**8.6x10**^**-36**^	0.47	6.8x10^-3^	0.87	0.21
Albumin (g/dL)	4.5±0.2	4.5 ± 0.4	4.4 ± 0.3	4.5 ± 0.4	4.3 ± 0.4	3.9±0.5	0.24	0.017	0.13	1.4x10^-3^	**2.2x10**^**-6**^
Total bilirubin (mg/dL)	0.7±0.3	0.9 ± 0.5	0.9±0.5	0.9±0.4	0.9±0.4	0.9±0.4	**2.2x10**^**-5**^	0.85	0.61	0.89	0.27
Cholinesterase (unit)	335.7±77.1	377.9±103.5	356.6±97.0	332.6±116.0	342.2±90.3	252.1±77.6	**1.8x10**^**-8**^	0.17	0.32	0.90	**7.6x10**^**-9**^
Type IV collagen 7S (ng/dL)	-	3.7±0.7	4.0±0.9	4.3±1.2	5.3±2.1	7.0±2.2	-	0.057	0.031	**2.2x10**^**-8**^	**1.9x10**^**-5**^
Hyaluronic acid (ng/dL)	-	27.6±25.3	31.6±27.9	50.3±71.6	82.5±86.2	160±114.5	-	0.18	0.11	**2.3x10**^**-5**^	**2.5x10**^**-6**^
Triglycerides (mg/dL)	97.1±67.1	157.3±87.0	153.1±84.3	162.0±94.0	161.3±83.9	126.0±55.7	**3.1x10**^**-23**^	0.66	0.49	0.78	2.1x10^-3^
Total cholesterol (mg/dL)	207.3±35.1	210.9±36.8	196.0±38.1	206.5±37.2	200.3±38.1	176.6±28.2	0.21	9.6x10^-4^	0.048	0.13	**1.1x10**^**-5**^
HbA1c (%)	5.4±0.5	6.1±1.2	5.8±1.0	6.1±1.4	6.2±1.2	6.2±1.3	**4.3x10**^**-6**^	0.21	0.24	0.26	0.22
IRI (μg/dL)	5.7±6.9	9.4±7.7	11.3±8.5	11.7±8.1	14.9±9.4	17.7±9.1	**7.9x10**^**-18**^	0.018	0.13	**2.8x10**^**-4**^	0.019
FPG (mg/dL)	90.8±12.5	113.0±34.7	105.5±25.6	111.8±27.2	113.7±33.4	126.7±30.1	**6.9x10**^**-25**^	0.12	0.015	0.94	**1.9x10**^**-4**^
HOMA-IR	1.2±1.0	2.4 ± 1.5	2.9 ± 2.4	3.4±2.3	4.2±2.9	5.7±3.9	**1.2x10**^**-23**^	0.19	0.11	1.1x10^-3^	4.2x10^-3^
hs-CRP (mg/dL)	865.6±2970.0	1316±2524.4	944.2±1180.6	1107.9±2062.6	1513.8±2267.4	2100.4±2508.4	**3.4x10**^**-5**^	0.89	0.82	0.016	0.11
Adiponectin (μg/mL)	-	7.2±3.4	6.4±2.9	7.0±3.3	6.6±3.3	8.6±4.3	-	0.092	0.22	0.28	0.032
Leptin (ng/mL)	-	9.8±7.0	9.5±8.1	11.8±8.6	13.6±10.5	14.5±18.3	-	0.43	0.056	0.25	0.5
Ferritin (ng/mL)	-	147.7±104.8	182.9±135.2	229.3±248.9	227.5±204.9	286.4±227.3	-	0.038	0.67	0.49	0.11
Uric acid (mg/dL)	-	5.9 ± 1.5	5.9 ± 1.2	5.7±1.6	5.7±1.5	5.4±1.5	-	0.59	0.41	0.94	0.32
PLT (x10^4^/μL)	23.4±5.2	22.9±5.5	22.9±5.5	22.7±6.6	19.7±6.1	14.9±6.4	0.27	0.90	0.43	**4.6x10**^**-5**^	**3.1x10**^**-8**^
ANA (0/1/2/3/4)	2403/1561 /527/32/128	51/24/7/1/1	58/27/7/1/2	38/15/7/1/0	198/115/41/12/16	12/8/2/3/3	0.090	0.97	0.93	0.11	0.29
Clinical history											
Diabetes (NGT/IGT/DM)	-	48/17/38	48/11/32	35/9/11	214/51/120	31/4/8		0.59**†**	0.15**†**	0.23**†**	0.11**†**
Hyperlipidemia (-/+)	4133/518	48/73	54/72	30/48	174/266	27/20	**3.6x10**^**-58**^**†**	0.70**†**	0.63**†**	0.95**†**	0.027**†**
Hypertension (-/+)	3806/845	77/44	69/57	43/35	219/222	20/32	**7.1x10**^**-7**^**†**	0.19**†**	0.92**†**	0.44**†**	0.17**†**
Liver biopsy features											
Brunt grade (1/2/3)	-	-	-	71/24/4	203/196/69	-	-	-	-	**1.7x10**^**-7**^	-
Brunt stage (1/2/3/4)	-	-	-	-	184/101/144/45	-	-	-	-	-	-
Fat droplet (1/2/3/4)	-	25/57/34/12	6/60/33/29	8/32/31/22	17/144/193/96	8/18/7/2	-	**3.2x10**^**-4**^	0.53	0.41	**2.5x10**^**-7**^
Iron deposition (0/1/2/3/4)	-	41/18/27/10/1	49/24/16/9/1	30/13/7/8/1	181/86/54/41/14	13/3/1/6/1	-	0.16	0.94	0.72	0.93

Measurements are shown as mean ± standard deviation. Categorical values are shown by the count number. The distribution of each class is compared to that immediately to its left. *The p*-values less than the threshold are in bold text. The threshold for significant association was set at 2.4x10^-3^ for the comparison between control and Matteoni type 1 and at 1.6x10^-3^ for the other comparisons. BMI: Body Mass Index, AST: Aspartate Aminotransferase, ALT: Alanine transaminase, GGT: Gamma-glutamyl transferase, IRI: Insulin, FPG: Fasting Plasma Glucose, HOMA-IR: Homeostasis model assessment-Insulin Resistance, PLT: Platelets, ANA: Antinuclear Antibodies. P-values are calculated by logistic regression otherwise stated:

†Chi-squared test.

### Genome-wide association studies

**Fig 1:**
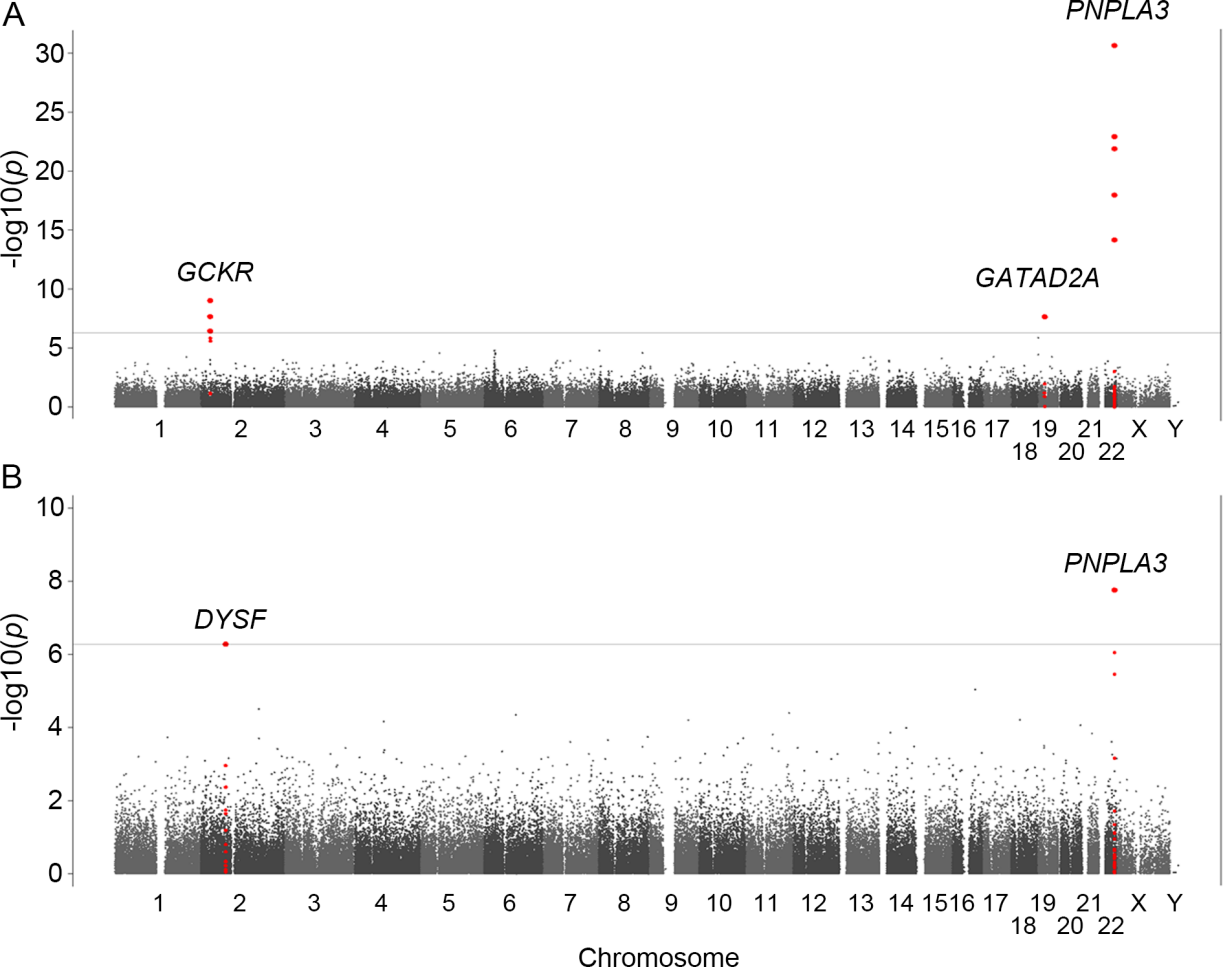
Manhattan plots of the GWA data. The *p*-values for genotyped SNPs from the GWA study for all NAFLD patients (A) and for NASH-HCC patients (B) compared to controls are plotted along each chromosome in −log_10_ scale. Horizontal line indicates the Bonferroni significance threshold (*p* = 5.3×10^−7^).

**Fig 2:**
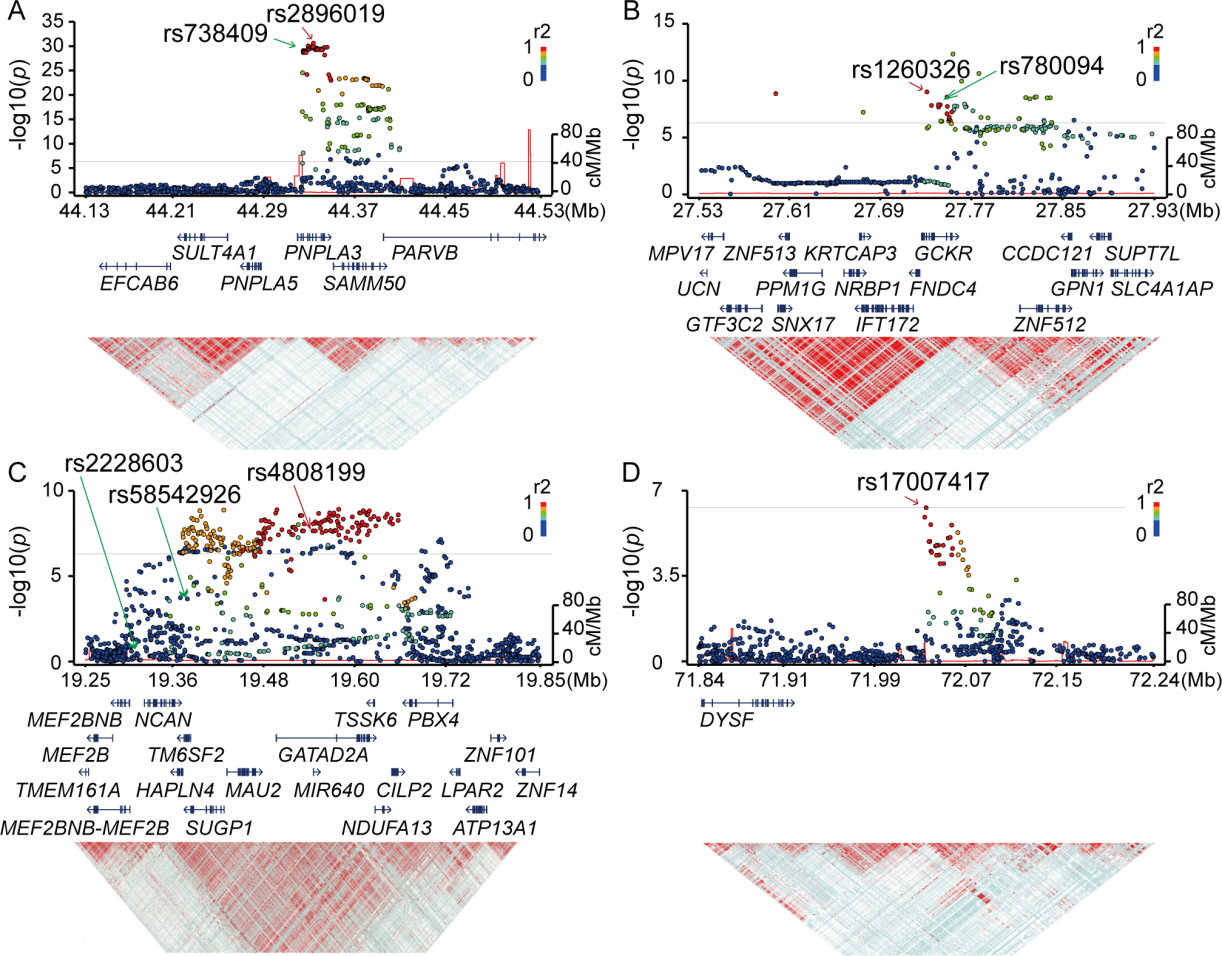
Regional Manhattan plots around the SNP markers showing genome-wide significance in the GWA studies. *P*-values, gene organization, and linkage disequilibrium (LD) plots according to the chromosomal position of the three significantly genome-wide associated regions for NAFLD (A-C) and the one region for NASH-HCC (D). Each figure spans 200 kb (A, B, and D) or 300 kb (C) in both the 5’ and 3’ directions from the SNP with the strongest association (shown with a red arrow) in the GWA studies. *P*-values are plotted for both genotyped and imputed SNPs in the upper panels, and previously reported SNPs with genome-wide significance are indicated by green arrows. The colors of the circles representing *p*-values correspond to the strength of LD (r2) from the most significant SNP in the GWA studies. The brightness of the red color in the LD plots in the lower panels also corresponds to the strength of LD.

We conducted a GWA study between 902 NAFLD patients including 58 NASH-HCC cases and 7,672 population controls for 93,606 SNP markers. A slight increase in *p*-values was observed after adjusting the population stratification using 10 PCs (λ = 1.12). Significant association signals (*p*<5.3x10^−7^) were detected in three chromosomes (Fig 1A, Table 2). The strongest association was observed for rs2896019 (*p* = 2.3x10^-31^), at 22q13.31 in the *PNPLA3* gene, which has repeatedly been reported as a strong genetic determinant for NAFLD. Rs738409, a non-synonymous SNP reportedly associated with NAFLD, was not identified in this analysis. However, its association with NAFLD was observed in the regional imputation analysis (*p* = 1.0x10^-29^) (Fig 2A). The second strongest association was detected for rs1260326 (*p* = 9.6x10^-10^), at 2p23.3 in the glucokinase regulator (*GCKR)* gene, another known susceptibility gene for NAFLD[12] (Fig 2B). A previously reported SNP, rs780094, also showed significant association with NAFLD (*p* = 2.1x10^-8^). The third strongest association was detected for rs4808199 (*p* = 2.3x10^-8^), in the vicinity of the GATA Zinc Finger Domain Containing 2A (*GATAD2A*) gene at 19p13.11. Rs4808199 was located in a 360-kb LD block encompassing *NCAN* and *TM6SF2*, which are both known to be associated with NAFLD[12,17] (Fig 2C). However, no association was detected for rs2228603 in *NCAN*, which was reported to be associated with NAFLD. The regional imputation analysis detected an indicative association signal for rs58542926 in *TM6SF2* (*p* = 2.2x10^-4^). A weak linkage disequilibrium between rs58542926 and rs4808199 was observed (r2 = 0.21.) The associations of *PNPLA3* and *GATAD2A* were lost when the type 1 to type 3 patients were used as cases. In contrast, they became stronger when only type 4 and NASH-HCC patients were used (*p* = 2.9x10^−34^ for rs2896019 and *p* = 2.0x10^−8^ for rs4808199, respectively), but the association of *GCKR* was lost (S2 Table).

**Table 2:** SNP markers showing significant association in the GWA studies.

dbSNPID	Chr.	Nearest Gene	Allele* (A1/A2)	Genotype counts** and frequency of A2 allele						NAFLD vs Control		NASH-HCC vs Control	
				NAFLD					Control	*p-*value	OR	*p-*value	OR
				Type 1	Type 2	Type 3	Type 4	NASH-HCC			(95%CI)		(95%CI)
rs2896019	22q13	*PNPLA3*	T/G	27/72/31	31/54/49	25/43/36	62/200/214	6/18/34	2259/3790/1621	**2.3x10**^**-31**^	**1.85**	**1.8x10**^**-8**^	**3.37**
				(0.52)	(0.57)	(0.55)	(0.66)	(0.74)	(0.46)		**(1.67–2.05)**		**(2.21–5.14)**
rs1260326	2p23	*GCKR*	C/T	17/65/48	19/54/61	9/47/48	72/210/194	9/16/33	1461/3666/2541	**9.6x10**^**-10**^	**1.38**	0.0031	1.84
				(0.62)	(0.66)	(0.68)	(0.63)	(0.71)	(0.57)		**(1.25–1.53)**		(1.23–2.75)
rs4808199	19p13	*GATAD2A*	G/A	72/43/15	61/57/16	58/36/10	208/216/52	21/33/4	4132/2990/544	**2.3x10**^**-8**^	**1.37**	0.020	1.59
				(0.28)	(0.33)	(0.27)	(0.34)	(0.35)	(0.27)		**(1.23–1.53)**		(1.08–2.35)
rs17007417	2p13	*DYSF*	C/T	85/40/5	99/34/1	79/23/2	329/136/11	25/25/8	5252/2207/197	0.47	0.95	**5.2x10**^**-7**^	**2.74**
				(0.19)	(0.13)	(0.13)	(0.17)	(0.35)	(0.17)		(0.83–1.09)		**(1.85–4.06)**

SNPs showing the strongest association in each chromosomal region are indicated.

*A2 represents the risk allele.

**Genotype counts are shown as (A1A1/A1A2/A2A2).

Odds ratio (OR) with 95% confidence interval (95%CI) was calculated for the risk allele. The *p*-values less than the threshold (*p* = 5.3×10^−7^) are in bold text.

We also performed a GWA study between 58 NASH-HCC patients and the same 7,672 controls. Population stratification was not observed (λ = 1.00) for this analysis. The significant association of *PNPLA3* was again observed (*p* = 1.8x10^-8^ for rs2896019). In addition, we detected a significant association in the vicinity of the dystrophy-associated fer-1-like protein (Dysferlin or *DYSF)* gene on 2p13.3 (*p* = 5.2x10^-7^ for rs17007417) (Fig 1B and Table 2). Regional imputation analysis detected an association peak consisting of multiple SNP markers, of which rs17007417 showed the highest association (Fig 2D). The list of SNP markers for which *p*<1.0x10^-5^ is provided as supplementary information (S3 Table) We also performed a GWA study using the ordinal logistic regression for Brunt grade as a metric for necroinflammatory activity, Brunt stage as the fibrosis stage, and fat-droplet content. No genome-wide significant SNPs were identified in these studies except for rs2896019 in *PNPLA3*, which showed a moderate association with fat-droplet content (p = 3.7x10^-4^) (S4 Table and S1 Fig).

### Associations of previously reported SNPs with NAFLD

Rs641738, a genetic variant that was recently shown to be associated with NAFLD[20], was included in the SNP markers examined in the current GWA study. However, we did not find any association of rs641738 with NAFLD, or with the Matteoni type 4 or NASH-HCC subgroup. We also examined the association of 14 other SNPs reviewed in Anstee et al. that were reported to be associated with the disease[28]. None of them showed genome-wide significance in our analysis, although rs58542926 in *TM6SF2* (p = 2.2x10^-4^) and rs1800234 in *PPARA* (p = 6.5x10^-5^) showed significant associations when the cut-off *p*-value was corrected for multiple testing to *p*<0.0035 (S5 Table). In addition, we examined whether the above 14 markers showed an association with the Brunt stage, Brunt grade, or fat-droplet content in the patient population. However, we did not find associations of any of the 14 markers with these NAFLD-related phenotypes.

### Impact of genetic variations on the pathogenicity of NAFLD

**Fig 3:**
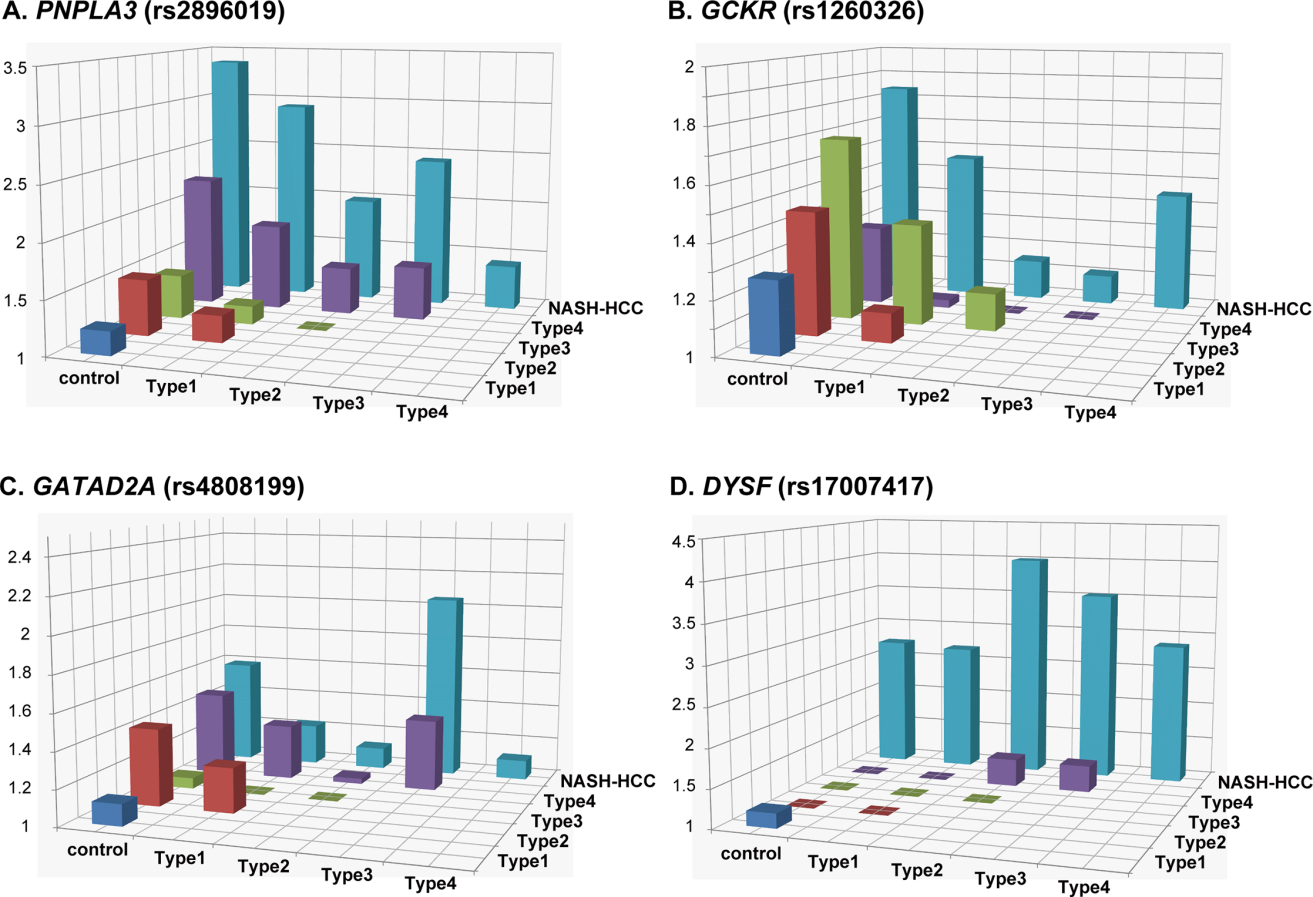
Histogram of the odds ratios for genotype distributions between different subgroups. Each box denotes the odds ratio (OR) comparing the control or patient subgroups shown on the horizontal axes. The *p*-values, ORs, and 95% confidence intervals are shown in the S6 Table.

We next investigated the impact of the genetic variations identified as significant in the GWA studies on the pathogenicity of the disease. The genotype distributions of rs2896019 in *PNPLA3*, rs4808199 in *GATAD2A*, rs1260326 in *GCKR*, and rs17007417 in *DYSF* were compared among the controls and the five patient subgroups. As reported previously[21], a significant difference (*p*<3.3x10^-3^) was observed for rs2896019 in Matteoni type 4 compared with the controls or the type 1 subgroup [*p* = 1.0x10^-28^ with an odds ratio (OR) of 2.23 and 95% confidence interval (95%CI) between 1.93 and 2.56, and *p* = 7.90x10^-6^, OR (95%CI) = 1.93 (1.45–2.58), respectively] (Fig 3A and S6A Table). The same trends were observed when type 4 was compared with type 2 and type 3, although the differences were not significant after correcting for multiple testing. The difference in rs2896019 was greatest between NASH-HCC and controls, type1, or type 3. The association was at the border of significance when NASH-HCC was compared with type 2 (*p* = 3.9x10^-3^), but more importantly, no association was observed when compared with type 4. Rs1260326 in *GCKR* showed significant associations with type 3, type 4, and NASH-HCC when compared with controls (Fig 3B and S6B Table). Notably, no statistical differences were observed among the five subgroups of patients. For rs4808199 in *GATAD2A*, only the comparison of type 4 and controls showed a significant difference (Fig 3C and S6C Table). Rs17007417 in *DYSF* was significantly different in NASH-HCC cases compared with controls or with any of the four Matteoni subgroups (Fig 3D and S6D Table).

### Risk estimation of NAFLD using genetic variations

**Fig 4:**
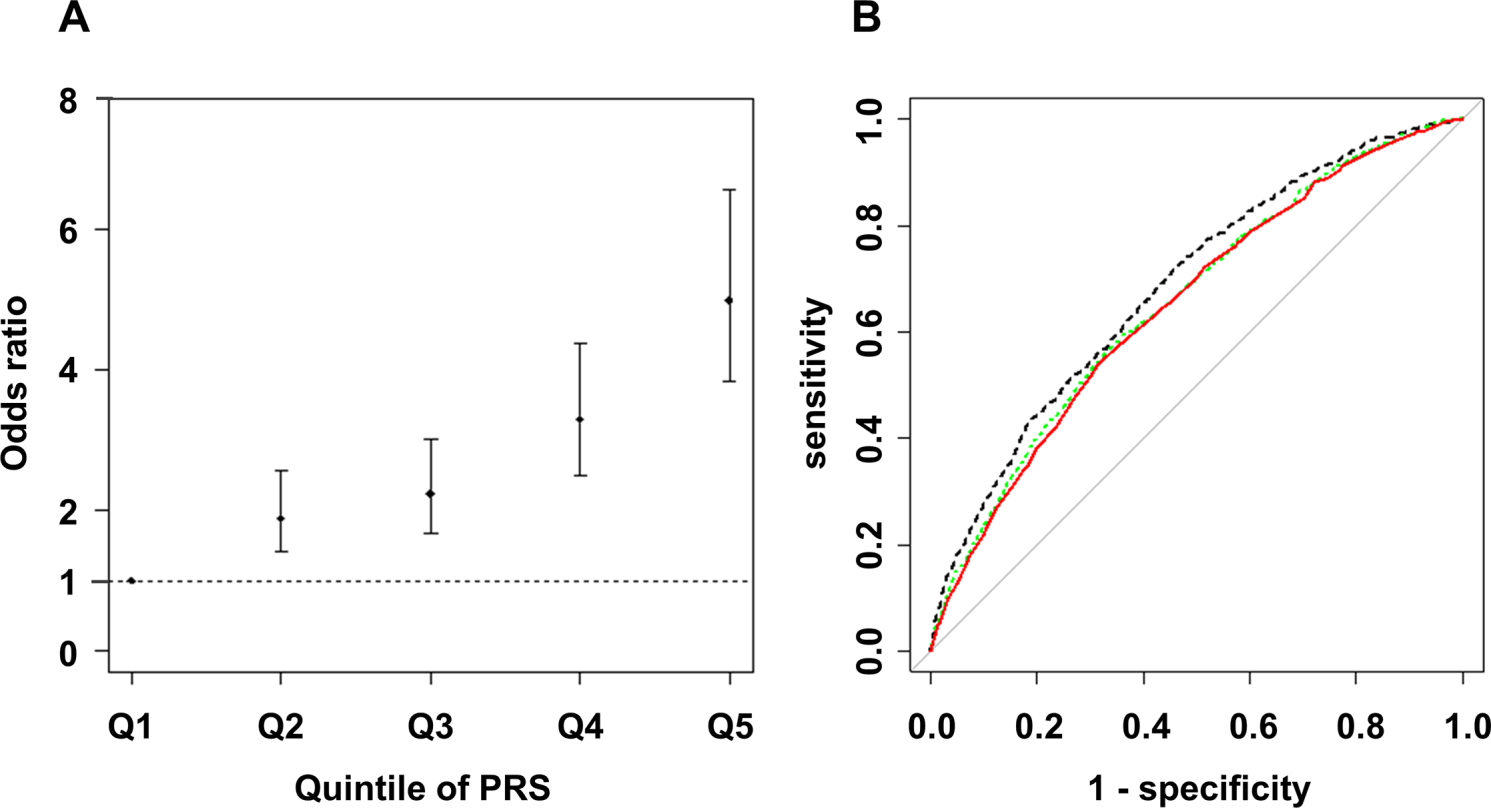
Risk estimation according to Polygenic Risk Scores for NAFLD patients compared with controls. The dot and bar denote the odds ratio (OR) and its 95% confidence interval in each quintile compared to the 1st quintile using rs2896019 in *PNPLA3*, rs1260326 in *GCKR*, and rs4808199 in *GATAD2A* (A), and the receiver operating characteristic (ROC) curve from the model (red line), and the ROC curves including previously reported SNPs (green dashed line) or candidate SNPs (p<1x10^-4^) identified in our GWA study (black dashed line) (B) are shown.

We next assessed the influence of risk alleles associated with NAFLD on the development of NAFLD. We first generated an estimated model by forward stepwise logistic regression using rs2896019 in *PNPLA3*, rs1260326 in *GCKR*, rs4808199 in *GATAD2A*, and rs17007417 in *DYSF*. Subsequently, rs2896019, rs1260326, and rs4808199 remained in the model. The ORs and 95%CIs compared with the 1st quintile PRS were 1.89 (1.40–2.58), 2.24 (1.68–3.01), 3.30 (2.51–4.38), and 5.00 (3.83–6.57) for the 2nd to 5th quintile PRS, respectively (Fig 4A). The area under the curve (AUC) was 0.65 (95% CI = 0.63–0.67). Next, the model was refined by including rs780094, rs738409, and rs58542926 instead of rs2896019, rs1260326, and rs4808199 and including 14 previously reported SNPs for NAFLD (S5 Table). Although rs56225452 in *SLC27A5*, rs1800234 in *PPARRA*, rs1799945 in *HFE*, and rs17883901 in *GCLC* were added to the model, the AUC was not improved (AUC = 0.65, 95%CI = 0.64–0.67). To estimate the possible maximum AUC for the current GWA study, we additionally included 10 candidate SNPs for which the p-value was <1x10^-4^ in our GWA study (S7 Table), and 13 total SNPs remained after the model selection. The AUC was increased to 0.69 (95%CI = 0.66–0.70) (Fig 4B). Risk estimation results for Matteoni type 1 to 3 vs. type 4 or NASH-HCC and for type 4 vs. NASH-HCC are shown in the S2 Fig and S8 Table.

## Discussion

*PNPLA3* is the strongest genetic determinant known for the development of NAFLD and NASH-HCC[6,12]. *PNPLA3* is a membrane protein located on the surface of lipid droplets. Rs738409, the I148M variant of *PNPLA3*, decreases triglyceride breakdown, leading to lipid retention in hepatocyte lipid droplets. The strong association of *PNPLA3* with type 4 and NASH-HCC but not with type 1 to type 3 indicates that it is involved in the later stages of NAFLD, particularly in liver fibrosis.

We identified *GATAD2A*, the function of which is not well understood, as a novel susceptibility gene for NAFLD. It is located in an LD block spanning *TM6SF2* and *NCAN*, which were previously reported as susceptible genes for NAFLD[12,17]. We also found that rs58542926 in *TM6SF2*, which had weak LD with rs4808199 in *GATAD2A*, showed a moderate association with NASH. Similar to *PNPLA3*, the association of *GATAD2A* was not observed with the type 1 to type 3 subgroups and was stronger with type 4 and NASH-HCC, indicating that it is related to the development of NASH. Rs4808199 in *GATAD2A* was previously shown to be strongly associated with the expression of *GATAD2A* and *MAU2*[22]. Furthermore, rs58542926 in *TM6SF2* and rs4808199 in *GATAD2A* are located in the same LD block, and genome-wide significant SNPs were also found in *TM6SF2*. Since there is accumulating biological evidence for the association between *TM6SF2* and NAFLD, the association of rs4808199 may be driven by the group of SNPs in high LD, which includes rs58542826. Given that there is no direct evidence that the nonsynonymous variant rs4808199 is causative, it is possible that other genetic variants functionally affect *TM6SF2*.

*GCKR* is an inhibitor of glucokinase (GCK), and its hepatic concentration is increased in NAFLD[29,30]. The risk allele of rs780094 in *GCKR* was shown to increase liver fat, possibly by increasing the expression of *C2orf16*[12]. However, no effect of rs780094 genotypes on *C2orf16* expression has been observed[22,31]. Rs780094 was also reported to be associated with the blood C reactive protein (CRP) level[32], but we did not observe this association using a high-sensitive CRP test (*p* = 0.77 by linear regression). Hence, it is still premature to draw clear conclusions about the mechanisms underlying the biological effects of this disease.

We identified an association peak at chromosome 2p13.3 in the GWA study using the NASH-HCC cases (Figs 1B and 2D). This SNP, rs17007417, was located 125-kb downstream of *DYSF* and was in an LD block encompassing a ‘gene desert’. *DYSF* is reported to be the causative gene for monogenic muscular disorders, such as muscular dystrophy, limb-girdle, type 2b, and Miyoshi muscular dystrophy 1. However, there is no report showing *DYSF* to be susceptible for multigenic diseases, including liver-related disorders. We also did not find an effect of rs17007417 on any gene expressions[22]. In addition, a limited number of NASH-HCC DNAs (58 samples) were included in the study due to the small number of biopsy-proven NASH-HCC patients, and this result was not validated using an independent sample set. Therefore, the involvement of rs17007417 in NASH-HCC still needs further investigation.

Rs641738 in *TMC4* located near *MBOAT7* on chromosome 19 did not show association with NAFLD and NASH-HCC in our Japanese study (S5 Table). While the association of rs641738 in patients of European descent was initially reported in 2015[19], the association has not been replicated in any other population to date. This may be due to the difference in LD patterns of the populations. According to the varLD[33], LD pattern between CEU and JPT population was significantly different within the *MBOAT7* (*p*<0.001) and *TMC4* (*p* = 0.002, p-values were calculated using 1000 genome phase3 dataset) regions. In addition, there are no large LD blocks in this region, so we could only successfully impute the SNP genotypes located near the genotyped SNPs (S3 Fig). These data suggest that rs641738 is not the causal variant, and in the European population the actual causal variant may lie close to and show high LD with rs641738. To confirm this hypothesis, a very dense genotyping or target sequencing in this region is necessary.

Risk estimation by PRS using the identified genome-wide significant SNPs for NAFLD clearly showed that the effect of the risk alleles, namely *PNPLA3*, *GATAD2A*, and *GCKR* was cumulative and increased the risk for NAFLD. Clinical and lifestyle information obtained from a prospective study might further improve this model.

The general population used as the control in this study could potentially include NAFLD patients. In addition, according to the 1000 Genomes Project phase 3 dataset[26], only 42.3% of the common SNPs in the Japanese population are tagged by the SNPs used in the current study with r2>0.5. These issues can lower the statistical power of the study and elicit false-negative results. In addition, even though almost all of the patient samples and all of the control samples were collected on Honshu island in Japan, some population stratification was observed, and none of our results were replicated in an independent cohort. Further confirmation of our findings is needed to draw conclusions with higher confidence.

The present study clearly demonstrated that genetic background exerted a marked influence on the severity of liver fibrosis and the development of NASH-HCC. We believe that the risk estimation using genetic markers will improve the accuracy of NAFLD diagnoses and help to guide treatment strategy decisions for patients.

## Supporting information

S1 Text SNP genotyping and quality controls. (DOCX)

S1 Table Summary of genotyping experiments. (DOCX)

S2 Table *P*-values of association studies comparing Types 1–3 with controls and comparing Type 4 and NASH-HCC with controls for the significantly associated SNPs identified in the GWA studies. (DOCX)

S3 Table List of the SNP markers showing *p*-values less than 1.0x10^-5^ in patient cases compared to general-population controls in the GWA studies. (DOCX)

S4 Table *P*-values and odds ratios of association for Brunt grade, Brunt stage, and fat-droplet content for the significantly associated SNPs identified in the GWA studies. (DOCX)

S5 Table *P*-values and odds ratios of the association studies for previously reported NAFLD associated SNPs. (DOCX)

S6 Table *P*-values and odds ratios for the genotype distribution between different subgroups. (DOCX)

S7 Table List of the SNP markers showing the lowest *p*-value with *p*<1.0x10^-4^ within each candidate locus identified in the GWA studies. (DOCX)

S8 Table Risk estimation according to Polygenic Risk Scores for NASH and NASH-HCC patients. (DOCX)

S1 Fig Manhattan plots for Brunt grade, Brunt stage, and fat-droplet content. (PPTX)

S2 Fig Risk estimation according to Polygenic Risk Scores for NASH and NASH-HCC patients. (PPTX)

S3 Fig Regional plots around the rs641738 in the study of all NAFLD patients compared to controls. (PPTX)
