# Supplementary material for: Risk estimation model for nonalcoholic fatty liver disease in the Japanese using multiple genetic markers
Source: PLoS One. 2018 Jan 31;13(1):e0185490. doi: 10.1371/journal.pone.0185490 (PMC5791941; doi:10.1371/journal.pone.0185490)
Supplement: S1 Text — (DOCX) [file pone.0185490.s001.docx]

# S1 Text. SNP genotyping and quality controls

Quality control of the genotyping results. All of the samples were subjected to standard quality controls, resulting in the exclusion of the following subjects: 1) one patient and 10 controls considered as non-Japanese by principle component analysis (PCA), 2) 32 patients and 637 controls showing kinship by PI-HAT, and 3) one patient and 45 controls with insufficient call rates (less than 0.95). Subsequently, quality controls were performed for 136,175 genotyped SNP markers that were common between the Illumina Human 610-Quad, Illumina Human Omni 2.5-8, and Illumina Infinium Core Exome arrays, and the following were excluded: 1) 33,438 SNPs with genotyping success rates less than 0.99, 2) 45 SNPs with a distorted Hardy–Weinberg equilibrium p-value (HWE: *P* < 1.0×10^-6^), and 3) 9,086 SNPs with a minor allele frequency (MAF) smaller than 0.01. Finally, 93,606 SNPs of 902 patient (844 NAFLD and 58 NASH-HCC) and 7,672 control samples were used for further analysis.
