# Supplementary material for: Risk estimation model for nonalcoholic fatty liver disease in the Japanese using multiple genetic markers
Source: PLoS One. 2018 Jan 31;13(1):e0185490. doi: 10.1371/journal.pone.0185490 (PMC5791941; doi:10.1371/journal.pone.0185490)
Supplement: S1 Table — (DOCX) [file pone.0185490.s002.docx]

# S1 Table. Summary of genotyping experiments.

|  | Cases | |  | Controls | | SNP genotyping array |
| --- | --- | --- | --- | --- | --- | --- |
|  | NAFLD | HCC |  | ACC | Nagahama |  |
| DNAs used for Kawaguchi *et al*.^17^ | 529 | - |  | 932 | - | 610K |
|  |  |  |  |  |  |  |
|  |  |  |  |  |  |  |
| Newly collected DNAs for the current study | - | 14 |  | - | 1614 | 610K |
|  | 349 | 44 |  | 2105 | 1618 | CoreExome |
|  |  |  |  |  | 1873 | 2.5M |
|  |  |  |  |  | 107 | 610K+CoreExome |
|  |  |  |  |  | 3 | 2.5M+CoreExome |
|  |  |  |  |  | 112 | 610K+2.5M+CoreExome |
| Total | 878 | 58 |  | 3037 | 5327 |  |
