# Supplementary material for: Risk estimation model for nonalcoholic fatty liver disease in the Japanese using multiple genetic markers
Source: PLoS One. 2018 Jan 31;13(1):e0185490. doi: 10.1371/journal.pone.0185490 (PMC5791941; doi:10.1371/journal.pone.0185490)
Supplement: S2 Table — (DOCX) [file pone.0185490.s003.docx]

# S2 Table. *P*-values of association studies comparing Type1-3 with control and comparing Type4 and NASH-HCC with control for the significantly associated SNPs in the GWA studies

| dbSNPID | Chr. | Nearest gene | Allele　(A1/A2) | Genotype counts and frequency of A2 allele | | |  | *p-*value and OR(95%CI) | |
| --- | --- | --- | --- | --- | --- | --- | --- | --- | --- |
|  |  |  |  | Type1-3 | Type4 and NASH-HCC | Control |  | type1-3  vs. Control | type4 and NASH-HCC vs. Control |
| rs2896019 | 22q13.31 | *PNPLA3* | T/G | 83/169/116 | 68/218/248 | 2259/3790/1621 |  | 3.7x10^-5^ 1.37 (1.18-1.60) | 2.9x10^-34^ 2.36 (2.01-2.76) |
|  |  |  |  | (0.54) | (0.67) | (0.46) |  |  |  |
| rs1260326 | 2p23.3 | *GCKR* | C/T | 45/166/157 | 81/226/227 | 1461/3666/2541 |  | 4.6x10^-6^ 1.44 (1.23-1.69) | 9.0x10^-6^ 1.35 (1.18-1.53) |
|  |  |  |  | (0.65) | (0.64) | (0.57) |  |  |  |
| rs4808199 | 19p13.11 | *GATAD2A* | G/A | 191/136/41 | 229/249/56 | 4132/2990/544 |  | 0.021 1.12 (1.03-1.44) | 2.0x10^-8^ 1.49 (1.29-1.69) |
|  |  |  |  | (0.30) | (0.34) | (0.27) |  |  |  |
| rs17007417 | 2p13.3 | *DYSF* | C/T | 263/97/8 | 354/161/19 | 5252/2207/197 |  | 0.37 1.10 (0.89-1.36) | 0.093 1.15 (1.-1.72) |
|  |  |  |  | (0.15) | (0.19) | (0.17) |  |  |  |
