# Supplementary material for: Risk estimation model for nonalcoholic fatty liver disease in the Japanese using multiple genetic markers
Source: PLoS One. 2018 Jan 31;13(1):e0185490. doi: 10.1371/journal.pone.0185490 (PMC5791941; doi:10.1371/journal.pone.0185490)
Supplement: S3 Table — (DOCX) [file pone.0185490.s004.docx]

# S3 Table. List of the SNP markers showing *p*-values less than 1.0x10^-5^ in patient cases compared to general-population controls in the GWA studies

|  | dbSNPID | Chr. | Position | Nearest gene | | Allele  (A1/A2) | Genotype counts and frequency of A2 allele | |  | Association | |
| --- | --- | --- | --- | --- | --- | --- | --- | --- | --- | --- | --- |
|  |  |  |  | Name | Location |  | Case | Control |  | *p*-value | OR (95%CI) |
| All patient cases | rs780094 | 2 | 27741237 | *GCKR* | intron | C/T | 127/404/371  (0.64) | 1475/3685/2511  (0.57) |  | 2.1x10^-8^ | 1.35  (1.21-1.49) |
|  | rs780092 | 2 | 27743154 | *GCKR* | intron | G/A | 62/303/537  (0.76) | 676/3137/3858  (0.71) |  | 3.4x10^-7^ | 1.35  (1.21-1.53) |
|  | rs1919127 | 2 | 27801493 | *C2orf16* | exon | T/C | 127/402/373  (0.64) | 1369/3745/2557  (0.58) |  | 1.9x10^-6^ | 1.29  (1.16-1.43) |
|  | rs1881396 | 2 | 27844601 | *ZNF512* | 3'UTR | G/T | 61/305/536  (0.76) | 641/3141/3889  (0.71) |  | 1.3x10^-6^ | 1.34  (1.19-1.50) |
|  | rs8731 | 2 | 27873326 | *GPN1* | 3'UTR | G/C | 200/480/222  (0.51) | 2249/3920/1502  (0.45) |  | 1.6x10^-6^ | 1.29  (1.16-1.43) |
|  | rs2668423 | 19 | 1370526 | *MUM1* | intron | G/T | 337/421/144  (0.39) | 3408/3382/875  (0.33) |  | 1.3x10^-6^ | 1.30  (1.16-1.43) |
|  | rs738491 | 22 | 44354111 | *SAMM50* | intron | C/T | 127/406/369  (0.63) | 1813/3786/2072  (0.52) |  | 1.2x10^-18^ | 1.60  (1.44-1.77) |
|  | rs2073082 | 22 | 44360007 | *SAMM50* | intron | A/G | 69/322/511  (0.75) | 908/3505/3258  (0.65) |  | 6.2x10^-15^ | 1.58  (1.41-1.77) |
|  | rs3761472 | 22 | 44368122 | *SAMM50* | exon | A/G | 175/435/292  (0.56) | 2534/3708/1429  (0.43) |  | 7.9x10^-24^ | 1.69  (1.52-1.87) |
|  | rs2143571 | 22 | 44391686 | *SAMM50* | intron | G/A | 176/433/293  (0.56) | 2475/3729/1467  (0.43) |  | 1.1x10^-22^ | 1.67  (1.50-1.85) |
| NASH-HCC | rs2143571 | 22 | 44391686 | *SAMM50* | intron | G/A | 7/24/27  (0.67) | 2472/3727/1466  (0.43) |  | 8.9x10^-7^ | 2.7  (1.82-4.00) |
|  | rs3761472 | 22 | 44368122 | *SAMM50* | exon | A/G | 8/24/26  (0.66) | 2536/3703/1429  (0.43) |  | 3.5x10^-6^ | 2.50  (1.69-3.70) |
