# Supplementary material for: Risk estimation model for nonalcoholic fatty liver disease in the Japanese using multiple genetic markers
Source: PLoS One. 2018 Jan 31;13(1):e0185490. doi: 10.1371/journal.pone.0185490 (PMC5791941; doi:10.1371/journal.pone.0185490)
Supplement: S4 Table — (DOCX) [file pone.0185490.s005.docx]

S4 Table. *P*-values and odds ratios of association for Brunt grade, Brunt stage, and fat-droplet content for the significantly associated SNPs identified in the GWA studies

| dbSNPID | Chr. | Nearest | Allele | Brunt stage | | Brunt grade | | fat droplet | |
| --- | --- | --- | --- | --- | --- | --- | --- | --- | --- |
|  |  | Gene | (A1/A2) | Odds ratio | p-value | Odds ratio | p-value | Odds ratio | p-value |
| rs2896019 | 22q13.31 | *PNPLA3* | T/G | 1.11 (0.91-1.35) | 0.29 | 1.27 (1.02-1.56) | 0.033 | 1.39 (1.17-1.67) | 3.7x10^-4^ |
| rs1260326 | 2p23.3 | *GCKR* | C/T | 0.95 (0.77-1.16) | 0.59 | 1.11 (0.89-1.37) | 0.36 | 1.03 (0.86-1.25) | 0.73 |
| rs4808199 | 19p13.11 | *GATAD2A* | G/A | 0.95 (0.77-1.17) | 0.62 | 0.98 (0.78-1.23) | 0.84 | 0.95 (0.78-1.15) | 0.60 |
| rs17007417 | 2p13.3 | *DYSF* | C/T | 1.03 (0.78-1.35) | 0.85 | 0.93 (0.70-1.24) | 0.63 | 0.71 (0.56-0.91) | 0.0062 |

Odds ratios and p-values were calculated by ordinal logistic regression. Odds ratios are calculated for A2.
