# Supplementary material for: Risk estimation model for nonalcoholic fatty liver disease in the Japanese using multiple genetic markers
Source: PLoS One. 2018 Jan 31;13(1):e0185490. doi: 10.1371/journal.pone.0185490 (PMC5791941; doi:10.1371/journal.pone.0185490)
Supplement: S5 Table — (DOCX) [file pone.0185490.s006.docx]

S5 Table. *P*-values and odds ratios of association studies for previously reported NAFLD-associated SNPs.

A. Association analyses between NAFLD population or the Matteoni type 4 or NASH-HCC subpopulation with controls

| dbSNPID | Chr. | Nearest  Gene | Allele  (A1/A2) | Genotype counts and frequency of A2 allele | | |  | *p-*value and OR (95%CI) | | Rsq | Pathway | Reference |
| --- | --- | --- | --- | --- | --- | --- | --- | --- | --- | --- | --- | --- |
|  |  |  |  | NAFLD | Type4 and  NASH-HCC | Control |  | NAFLD vs Control | Type4  and NASH-HCC vs Control |  |  |  |
| rs13412852 | 2p25.1 | *LPIN1* | C/T | 522/339/39 | 302/211/21 | 4477/2793/401 |  | 0.76 | 0.85 | 0.60 | Lipid metabolism | 1 |
|  |  |  |  | (0.23) | (0.24) | (0.23) |  | 0.98 (0.87-1.10) | 1.02 (0.88-1.18) |  |  |  |
| rs1800591 | 4q23 | *MTTP* | G/T | 627/245/28 | 360/159/15 | 5482/1999/190 |  | 0.17 | 0.058 | 0.99 | Lipid metabolism | 2 |
|  |  |  |  | (0.17) | (0.18) | (0.16) |  | 1.10 (0.96-1.25) | 1.17 (1.00-1.38) |  |  |  |
| rs56225452 | 19q13.43 | *SLC27A5* | T/C | 1/36/863 | 1/16/517 | 7/450/7214 |  | 0.029 | 0.014 | 0.57 | Lipid metabolism | 3 |
|  |  |  |  | (0.98) | (0.98) | (0.97) |  | 1.45 (1.04-2.02) | 1.82 (1.13-2.92) |  |  |  |
| rs7946 | 17p11.2 | *PEMT* | T/C | 463/356/81 | 264/216/54 | 3961/3112/598 |  | 0.53 | 0.11 | 0.99 | Lipid metabolism | 4 |
|  |  |  |  | (0.29) | (0.30) | (0.28) |  | 0.97 (0.87-1.08) | 1.12 (0.97-1.28) |  |  |  |
| rs58542926 | 19p13.11 | *TM6SF2* | C/T | 736/153/13 | 430/98/6 | 6543/1090/38 |  | **2.2x10^-4^** | **7.4x10^-4^** | 0.92 | Lipid metabolism | 5 |
|  |  |  |  | (0.10) | (0.10) | (0.076) |  | **1.39 (1.16-1.64)** | **1.45 (1.16-1.79)** |  |  |  |
| rs641738 | 19q13.42 | *MBOAT7* | C/T | 540/320/41 | 321/184/29 | 4682/2617/372 |  | 0.73 | 0.54 | Genotyped | Lipid metabolism | 6 |
|  |  |  |  | (0.22) | (0.23) | (0.22) |  | 1.02 (0.91-1.15) | 1.05 (0.90-1.22) |  |  |  |
| rs1800234 | 22q13.31 | *PPARA* | C/T | 0/37/863 | 0/20/514 | 14/518/7139 |  | **6.5x10^-5^** | **7.2x10^-4^** | 0.71 | Lipid metabolism | 7,8 |
|  |  |  |  | (0.98) | (0.98) | (0.96) |  | **1.98 (1.42-2.77)** | **2.17 (1.39-3.40)** |  |  |  |
| rs1800629 | 6p21.33 | *TNF* | G/A | 877/22/1 | 518/15/1 | 7534/135/2 |  | 0.87 | 0.39 | 0.94 | Immune response | 9,10 |
|  |  |  |  | (0.013) | (0.016) | (0.0090) |  | 1.04 (0.68-1.58) | 1.24 (0.76-2.03) |  |  |  |
| rs1799945 | 6p22.2 | *HFE* | G/C | 882/18/0 | 0/13/521 | 9/414/7248 |  | 0.061 | 0.37 | 0.98 | Oxidative stress | 11 |
|  |  |  |  | (0.99) | (0.99) | (0.97) |  | 1.58 (0.98-2.54) | 1.29 (0.74-2.26) |  |  |  |
| rs17883901 | 6p12.1 | *GCLC* | G/A | 641/233/26 | 389/132/13 | 5703/1814/154 |  | 0.023 | 0.38 | 0.64 | Oxidative stress | 12 |
|  |  |  |  | (0.16) | (0.15) | (0.14) |  | 1.17 (1.02-1.33) | 1.08 (0.91-1.29) |  |  |  |
| rs4880 | 6q25.3 | *SOD2* | A/G | 674/217/9 | 399/131/4 | 5892/1667/112 |  | 0.35 | 0.51 | 0.99 | Oxidative stress | 2 |
|  |  |  |  | (0.13) | (0.13) | (0.12) |  | 1.07 (0.93-1.24) | 1.07 (0.88-1.28) |  |  |  |
| rs762623 | 6p21.2 | *CDKN1A* | G/A | 522/339/39 | 302/211/21 | 4477/2793/401 |  | 0.85 | 0.96 | 0.67 | Tumor | 13 |
|  |  |  |  | (0.23) | (0.24) | (0.23) |  | 1.01 (0.87-1.18) | 1.00 (0.82-1.20) |  |  |  |
| rs3750861 | 10p15.1 | *KLF6* | C/T | 627/245/28 | 360/159/15 | 5482/1999/190 |  | 0.47 | 0.81 | 0.85 | Tumor | 14 |
|  |  |  |  | (0.17) | (0.18) | (0.16) |  | 0.89 (0.65-1.21) | 1.05 (0.70-1.57) |  |  |  |
| rs1044498 | 6q23.2 | *ENPP1* | A/C | 1/36/863 | 1/16/517 | 7/450/7214 |  | 0.83 | 0.74 | 0.99 | Glucose metabolism and insulin resistance | 15 |
|  |  |  |  | (0.98) | (0.98) | (0.97) |  | 0.98 (0.83-1.16) | 1.04 (0.84-1.28) |  |  |  |

Rsq : r-square as imputation quality calculated by MACH. Odds ratios are calculated for A2. Statistical significance was set at p<0.0035.

B. Association analyses for Brunt stage, Brunt grade, and fat droplet contents

| dbSNPID | Chr. | Nearest | Allele | Brunt stage | | Brunt grade | | Fat droplet | |
| --- | --- | --- | --- | --- | --- | --- | --- | --- | --- |
|  |  | Gene | (A1/A2) | Odds ratio | p-value | Odds ratio | p-value | Odds ratio | p-value |
| rs13412852 | 2p25 | *LPIN1* | C/T | 1.14 (0.90-1.46) | 0.27 | 0.93 (0.71-1.20) | 0.57 | 0.99 (0.79-1.23) | 0.91 |
| rs1800591 | 4q23 | *MTTP* | T/G | 0.84 (0.65-1.09) | 0.2 | 0.85 (0.65-1.12) | 0.25 | 0.85 (0.67-1.08) | 0.19 |
| rs56225452 | 19q13 | *SLC27A5* | T/C | 1.30 (0.65-2.63) | 0.45 | 1.21 (0.56-2.59) | 0.63 | 1.50 (0.79-2.85) | 0.21 |
| rs7946 | 17p11 | *PEMT* | T/C | 0.80 (0.65-0.99) | 0.042 | 0.77 (0.61-0.97) | 0.024 | 0.93 (0.77-1.14) | 0.49 |
| rs58542926 | 19p13.11 | *TM6SF2* | C/T | 1.17(0.84-1.63) | 0.34 | 1.08 (0.75-1.53) | 0.89 | 1.08 (0.78-1.53) | 0.62 |
| rs641738 | 19q13 | *MBOAT7* | C/T | 1.08 (0.85-1.39) | 0.41 | 0.90 (0.69-1.16) | 0.41 | 1.23 (0.97-1.54) | 0.075 |
| rs1800234 | 22q.13 | *PPARA* | C/T | 0.99 (0.39-2.54) | 0.99 | 0.70 (0.25-1.98) | 0.51 | 1.09 (0.51-2.34) | 0.83 |
| rs1800629 | 6p21 | *TNF* | A/G | 0.30 (0.09-0.99) | 0.047 | 0.62 (0.13-3.00) | 0.55 | 3.98 (1.22-13.01) | 0.022 |
| rs1799945 | 6p22 | *HFE* | G/C | 0.53 (0.21-1.34) | 0.18 | 0.37 (0.14-0.97) | 0.044 | 1.08 (0.47-2.48) | 0.86 |
| rs17883901 | 6p12 | *GCLC* | A/G | 1.20 (0.92-1.58) | 0.18 | 1.06 (0.80-1.42) | 0.68 | 0.86 (0.67-1.09) | 0.22 |
| rs4880 | 6q25 | *SOD2* | A/G | 0.70 (0.51-0.97) | 0.03 | 0.86 (0.61-1.20) | 0.38 | 0.91 (0.69-1.20) | 0.49 |
| rs762623 | 6p21 | *CDKN1A* | G/A | 0.88 (0.65-1.17) | 0.37 | 0.96 (0.70-1.30) | 0.78 | 0.90 (0.69-1.18) | 0.44 |
| rs3750861 | 10p15 | *KLF6* | C/T | 0.97 (0.50-1.88) | 0.93 | 1.29 (0.62-2.66) | 0.49 | 1.37 (0.77-2.43) | 0.29 |
| rs1044498 | 6q23 | *ENPP1* | C/A | 1.11 (0.78-1.57) | 0.56 | 1.08 (0.74-1.58) | 0.68 | 0.69 (0.51-0.94) | 0.019 |

Odds ratios are calculated for A2. Statistically significant p-value was set at p<0.0035.

1. Valenti, L. *et al.* LPIN1 rs13412852 polymorphism in pediatric nonalcoholic fatty liver disease. *J. Pediatr. Gastroenterol. Nutr.* **54,** 588–593 (2012).

2. Namikawa, C. *et al.* Polymorphisms of microsomal triglyceride transfer protein gene and manganese superoxide dismutase gene in non-alcoholic steatohepatitis. *J. Hepatol.* **40,** 781–786 (2004).

3. Auinger, A. *et al.* A promoter polymorphism in the liver-specific fatty acid transport protein 5 is associated with features of the metabolic syndrome and steatosis. *Horm. Metab. Res. Horm. Stoffwechselforschung Horm. Metab.* **42,** 854–859 (2010).

4. Song, J. *et al.* Polymorphism of the PEMT gene and susceptibility to nonalcoholic fatty liver disease (NAFLD). *FASEB J. Off. Publ. Fed. Am. Soc. Exp. Biol.* **19,** 1266–1271 (2005).

5. Kozlitina, J. *et al.* Exome-wide association study identifies a TM6SF2 variant that confers susceptibility to nonalcoholic fatty liver disease. *Nat. Genet.* **46,** 352–356 (2014).

6. Buch, S. *et al.* A genome-wide association study confirms PNPLA3 and identifies TM6SF2 and MBOAT7 as risk loci for alcohol-related cirrhosis. *Nat. Genet.* **47,** 1443–1448 (2015).

7. Chen, S., Li, Y., Li, S. & Yu, C. A Val227Ala substitution in the peroxisome proliferator activated receptor alpha (PPAR alpha) gene associated with non-alcoholic fatty liver disease and decreased waist circumference and waist-to-hip ratio. *J. Gastroenterol. Hepatol.* **23,** 1415–1418 (2008).

8. Dongiovanni, P. *et al.* Lack of association between peroxisome proliferator-activated receptors alpha and gamma2 polymorphisms and progressive liver damage in patients with non-alcoholic fatty liver disease: a case control study. *BMC Gastroenterol.* **10,** 102 (2010).

9. Valenti, L. *et al.* Tumor necrosis factor α promoter polymorphisms and insulin resistance in nonalcoholic fatty liver disease. *Gastroenterology* **122,** 274–280 (2002).

10. Tokushige, K. *et al.* Influence of TNF gene polymorphisms in Japanese patients with NASH and simple steatosis. *J. Hepatol.* **46,** 1104–1110 (2007).

11. George, D. K. *et al.* Increased hepatic iron concentration in nonalcoholic steatohepatitis is associated with increased fibrosis. *Gastroenterology* **114,** 311–318 (1998).

12. Oliveira, C. P. M. S. *et al.* Association of polymorphisms of glutamate-cystein ligase and microsomal triglyceride transfer protein genes in non-alcoholic fatty liver disease. *J. Gastroenterol. Hepatol.* **25,** 357–361 (2010).

13. Aravinthan, A. *et al.* Gene polymorphisms of cellular senescence marker p21 and disease progression in non-alcohol-related fatty liver disease. *Cell Cycle* **13,** 1489–1494 (2014).

14. Miele, L. *et al.* The Kruppel-like factor 6 genotype is associated with fibrosis in nonalcoholic fatty liver disease. *Gastroenterology* **135,** 282–291.e1 (2008).

15. Dongiovanni, P. *et al.* Genetic variants regulating insulin receptor signalling are associated with the severity of liver damage in patients with non-alcoholic fatty liver disease. *Gut* **59,** 267–273 (2010).
