# Supplementary material for: Risk estimation model for nonalcoholic fatty liver disease in the Japanese using multiple genetic markers
Source: PLoS One. 2018 Jan 31;13(1):e0185490. doi: 10.1371/journal.pone.0185490 (PMC5791941; doi:10.1371/journal.pone.0185490)
Supplement: S6 Table — (DOCX) [file pone.0185490.s007.docx]

# S6 Table. *P*-values and odds ratios for genotype distribution between different subgroups.

**S6A. rs2896019 (*PNPLA3*)**

|  | **control** | **type1** | **type2** | **type3** | **type4** |
| --- | --- | --- | --- | --- | --- |
| **NASH-HCC** | **1.8x10^-8^** | **1.3x10^-4^** | 3.9x10^-3^ | **7.8x10^-4^** | 0.11 |
|  | **3.37** | **2.93** | 1.99 | 2.43 | 1.42 |
|  | **(2.21-5.14)** | **(1.69-5.08)** | (1.25-3.16) | (1.45-4.09) | (0.92-2.19) |
| **type4** | **1.0x10^-28^** | **3.6x10^-5^** | 8.1x10^-3^ | 7.1x10^-3^ |  |
|  | **2.23** | **1.81** | 1.44 | 1.5 |  |
|  | **(1.93-2.56)** | **(1.37-2.4)** | (1.1-1.89) | (1.12-2.02) |  |
| **type3** | 0.014 | 0.43 | 0.58 |  |  |
|  | 1.41 | 1.17 | 0.91 |  |  |
|  | (1.07-1.86) | (0.8-1.7) | (0.64-1.28) |  |  |
| **type2** | **8.6x10^-4^** | 0.22 |  |  |  |
|  | **1.52** | 1.25 |  |  |  |
|  | **(1.19-1.94)** | (0.88-1.78) |  |  |  |
| **type1** | 0.11 |  |  |  |  |
|  | 1.22 |  |  |  |  |
|  | (0.96-1.57) |  |  |  |  |

**S6B rs1260326 (*GCKR*)**

|  | **control** | **type1** | **type2** | **type3** | **type4** |
| --- | --- | --- | --- | --- | --- |
| **NASH-HCC** | **3.1x10^-3^** | 0.084 | 0.56 | 0.68 | 0.082 |
|  | **1.84** | 1.56 | 1.15 | 1.11 | 1.45 |
|  | **(1.23-2.75)** | (0.94-2.57) | (0.72-1.82) | (0.67-1.86) | (0.95-2.21) |
| **type4** | **2.0x10^-4^** | 0.82 | 0.34 | 0.15 |  |
|  | **1.3** | 1.03 | 0.87 | 0.79 |  |
|  | **(1.13-1.49)** | (0.78-1.37) | (0.66-1.16) | (0.57-1.09) |  |
| **type3** | **5.1x10^-4^** | 0.13 | 0.50 |  |  |
|  | **1.69** | 1.38 | 1.14 |  |  |
|  | **(1.26-2.28)** | (0.91-2.09) | (0.77-1.7) |  |  |
| **type2** | 3.3x10^-3^ | 0.56 |  |  |  |
|  | 1.46 | 1.11 |  |  |  |
|  | (1.13-1.89) | (0.77-1.61) |  |  |  |
| **type1** | 0.068 |  |  |  |  |
|  | 1.27 |  |  |  |  |
|  | (0.98-1.63) |  |  |  |  |

**S6C. rs4808199 (*GATAD2A*)**

|  | **control** | **type1** | **type2** | **type3** | **type4** |
| --- | --- | --- | --- | --- | --- |
| **NASH-HCC** | 0.020 | 0.42 | 0.64 | 0.017 | 0.62 |
|  | 1.59 | 1.23 | 1.12 | 2.05 | 1.11 |
|  | (1.08-2.35) | (0.75-2.00) | (0.69-1.84) | (1.14-3.69) | (0.73-1.69) |
| **type4** | **2.1x10^-7^** | 0.084 | 0.86 | 0.054 |  |
|  | **1.47** | 1.31 | 1.03 | 1.4 |  |
|  | **(1.27-1.70)** | (0.96-1.77) | (0.77-1.38) | (0.99-1.97) |  |
| **type3** | 0.71 | 0.95 | 0.12 |  |  |
|  | 1.06 | 0.99 | 0.73 |  |  |
|  | (0.77-1.46) | (0.66-1.47) | (0.48-1.09) |  |  |
| **type2** | 7.6x10^-3^ | 0.23 |  |  |  |
|  | 1.43 | 1.25 |  |  |  |
|  | (1.1-1.87) | (0.86-1.82) |  |  |  |
| **type1** | 0.42 |  |  |  |  |
|  | 1.12 |  |  |  |  |
|  | (0.85-1.48) |  |  |  |  |

**S6D. rs17007417 (*DYSF*)**

|  | **control** | **type1** | **type2** | **type3** | **type4** |
| --- | --- | --- | --- | --- | --- |
| **NASH-HCC** | **5.2x10^-7^** | **5.9x10^-4^** | **9.0x10^-6^** | **7.7x10^-5^** | **7.5x10^-6^** |
|  | **2.74** | **2.69** | **4.00** | **3.53** | **2.87** |
|  | **(1.85-4.06)** | **(1.53-4.72)** | **(2.17-7.37)** | **(1.89-6.6)** | **(1.81-4.56)** |
| **type4** | 0.98 | 0.31 | 0.14 | 0.20 |  |
|  | 1.00 | 0.83 | 1.36 | 1.35 |  |
|  | (0.84-1.2) | (0.58-1.19) | (0.9-2.04) | (0.86-2.12) |  |
| **type3** | 0.16 | 0.11 | 0.85 |  |  |
|  | 0.75 | 0.65 | 0.95 |  |  |
|  | (0.5-1.13) | (0.39-1.1) | (0.53-1.68) |  |  |
| **type2** | 0.17 | 0.23 |  |  |  |
|  | 0.78 | 0.73 |  |  |  |
|  | (0.54-1.11) | (0.44-1.22) |  |  |  |
| **type1** | 0.28 |  |  |  |  |
|  | 1.19 |  |  |  |  |
|  | (0.87-1.63) |  |  |  |  |
