# Supplementary material for: Risk estimation model for nonalcoholic fatty liver disease in the Japanese using multiple genetic markers
Source: PLoS One. 2018 Jan 31;13(1):e0185490. doi: 10.1371/journal.pone.0185490 (PMC5791941; doi:10.1371/journal.pone.0185490)
Supplement: S7 Table — (DOCX) [file pone.0185490.s008.docx]

# S7 Table. List of the SNP markers showing the lowest *p*-value with *p*<1.0x10^-4^ within each candidate locus identified in the GWA studies

| Case/Control | dbSNPID | Chr. | Position | Nearest gene | | Allele (A1/A2) | Genotype counts and frequency of A2 allele | |  | Association | |
| --- | --- | --- | --- | --- | --- | --- | --- | --- | --- | --- | --- |
|  |  |  |  | Name | Location |  | Case | Control |  | *p*-value | OR (95%CI) |
| NAFLD  /Control | rs3756498 | 5 | 52778210 | *FST* | intron | A/G | 18/237/647  (0.85) | 267/2228/5168  (0.82) |  | 2.7x10^-5^ | 1.34  (1.17-1.55) |
|  | rs259940 | 6 | 30011934 | *ZNRD1ASP* | intron | A/G | 298/435/169  (0.43) | 2751/3697/1218  (0.40) |  | 1.6x10^-5^ | 1.27  (1.14-1.42) |
|  | rs9267845 | 6 | 32193698 | *NOTCH4* | intergenic | T/A | 37/266/599  (0.81) | 357/2551/4751  (0.79) |  | 2.6x10^-5^ | 1.23  (1.12-1.37) |
|  | rs893875 | 8 | 2011098 | *MYOM2* | intron | G/A | 4916/2457/293  (0.20) | 648/228/26  (0.16) |  | 1.7x10^-5^ | 1.35  (1.12-1.37) |
|  | rs2980876 | 8 | 126481694 | *TRIB* | intergenic | G/A | 343/414/145  (0.39) | 3416/3395/854  (0.33) |  | 2.7x10^-5^ | 1.25  (1.12-1.39) |
|  | rs12871748 | 13 | 69782361 | *LOC100128625* | intergenic | T/C | 154/446/301  (0.58) | 1636/3850/2179  (0.54) |  | 7.1x10^-5^ | 1.26  (1.13-1.37) |
|  | rs4773424 | 13 | 89635132 | *LOC100131026* | intergenic | G/A | 484/348/70  (0.27) | 4555/2709/402  (0.23) |  | 5.8x10^-5^ | 1.26  (1.13-1.42) |
|  | rs315499 | 17 | 30021321 | *MIR365-2* | intergenic | A/G | 222/476/204  (0.49) | 2312/3799/1536  (0.45) |  | 5.7x10^-5^ | 1.23  (1.13-1.37) |
|  | rs2668423 | 19 | 1370526 | *MUM1* | intron | G/T | 337/421/144  (0.39) | 3408/3382/875  (0.34) |  | 1.3x10^-6^ | 1.30  (1.16-1.43) |
|  | rs8100045 | 19 | 40162019 | *LOC400696* | intergenic | C/T | 528/317/57  (0.24) | 4882/2478/301  (0.20) |  | 5.7x10^-5^ | 1.28  (1.13-1.37) |
| Matteoni type 4 + NASH-HCC/ Matteoni type 1-3 | rs9396195 | 6 | 56163121 | *LOC100288495* | intergenic | G/A | 423/102/7  (0.11) | 330/37/1  (0.053) |  | 5.8 x10^-5^ | 2.18  (1.49-3.18) |
|  | rs620806 | 7 | 105339137 | *ATXN7L1* | intron | G/A | 29/236/267  (0.72) | 53/166/149  (0.63) |  | 3.3 x10^-5^ | 1.56  (1.27-1.93) |
|  | rs3133278 | 8 | 105474935 | *DPYS* | intron | T/C | 214/259/59  (0.35) | 197/149/22  (0.26) |  | 1.4 x10^-5^ | 1.62  (1.30-2.01) |
|  | rs4763351 | 12 | 13686475 | *GRIN2B* | intergenic | T/C | 106/266/160 (0.55) | 107/184/77  (0.46) |  | 6.9 x10^-5^ | 1.48  (1.22-1.79) |
|  | rs3803064 | 12 | 113173494 | *RPH3A* | intergenic | G/A | 40/218/274  (0.72) | 47/175/146  (0.64) |  | 5.7 x10^-5^ | 1.54  (1.25-1.90) |
|  | rs3888264 | 16 | 58006269 | *CNGB1* | intergenic | C/A | 5/91/436  (0.91) | 7/104/257  (0.84) |  | 3.1 x10^-5^ | 1.86  (1.39-2.50) |
|  | rs7198400 | 16 | 78431277 | *WWOX* | intron | T/G | 70/236/225  (0.65) | 112/181/75  (0.55) |  | 6.2 x10^-5^ | 1.49  (1.22-1.80) |
|  | rs1990287 | 17 | 26819065 | *SLC13A2* | intron | A/G | 6/83/442  (0.91) | 4/97/267  (0.857) |  | 8.2 x10^-5^ | 1.85  (1.36-2.51) |
|  | rs4801135 | 18 | 52783138 | *TCF4* | intergenic | T/C | 12/136/384  (0.85) | 21/131/216  (0.77) |  | 1.1 x10^-5^ | 1.73  (1.35-2.20) |
|  | rs381716 | 21 | 41235752 | *PCP4* | intergenic | A/G | 176/258/98  (0.43) | 167/156/45  (0.33) |  | 4.5 x10^-5^ | 1.51  (1.24-1.84) |
| NASH-HCC /Matteoni type4 | rs6594492 | 5 | 110355405 | *LOC100131280* | intergenic | G/A | 14/29/14  (0.50) | 207/229/39  (0.32) |  | 4.9 x10^-5^ | 2.41  (1.58-3.68) |
|  | rs2944529 | 10 | 132958926 | *TCERG1L* | intron | G/A | 19/28/10  (0.42) | 268/185/22  (0.24) |  | 9.8 x10^-5^ | 2.39  (1.54-3.70) |
|  | rs10142295 | 14 | 30600423 | *RPS6P24* | intergenic | T/C | 21/22/14  (0.44) | 267/181/27  (0.25) |  | 1.8 x10^-5^ | 2.49  (1.64-3.79) |
|  | rs4129156 | 18 | 25437752 | *CDH2* | intergenic | T/C | 22/25/10  (0.40) | 312/136/26  (0.20) |  | 8.6 x10^-5^ | 2.24  (1.50-3.35) |

Odds ratios were calculated for A2.
