# Supplementary material for: Risk estimation model for nonalcoholic fatty liver disease in the Japanese using multiple genetic markers
Source: PLoS One. 2018 Jan 31;13(1):e0185490. doi: 10.1371/journal.pone.0185490 (PMC5791941; doi:10.1371/journal.pone.0185490)
Supplement: S8 Table — (DOCX) [file pone.0185490.s009.docx]

# S8 Table. Risk estimation according to Polygenic Risk Scores for NASH and NASH-HCC patients.

| Statistical test | | SNPs* | AUC  (95%CI) | Quintile | OR  (95% CI) |
| --- | --- | --- | --- | --- | --- |
| Case | Control |  |  |  |  |
| Type 4 and NASH-  HCC | Type 1-3 | rs2896019 (*PNPLA3*),  rs4808199 (*GATAD2A*)  rs17007417 (*DYSF*) | 0.63  (0.59-0.67) | 5th | 4.39  (2.66-7.39) |
|  |  |  |  | 4th | 2.01  (1.33-3.04) |
|  |  |  |  | 3rd | 1.77  (1.15-2.74) |
|  |  |  |  | 2nd | 1.29  (0.82-1.96) |
|  |  |  |  | 1st | Reference |
| NASH-  HCC | Type 4 | rs2896019 (*PNPLA3*),  rs17007417 (*DYSF*) | 0.72  (0.64-0.79) | 5th | 15.86  (3.66-144.33) |
|  |  |  |  | 4th | 6.35  (1.33-60.54) |
|  |  |  |  | 3rd | 4.30  (0.83-42.48) |
|  |  |  |  | 2nd | 3.35  (0.62-33.82) |
|  |  |  |  | 1st | Reference |

*SNPs included in the model constructed using genome-wide significant SNPs for PRS (red line in S2 Fig).
