# Supplementary material for: Risk estimation model for nonalcoholic fatty liver disease in the Japanese using multiple genetic markers
Source: PLoS One. 2018 Jan 31;13(1):e0185490. doi: 10.1371/journal.pone.0185490 (PMC5791941; doi:10.1371/journal.pone.0185490)
Supplement: S1 Fig — (PPTX) [file pone.0185490.s010.pptx]

## Slide 1
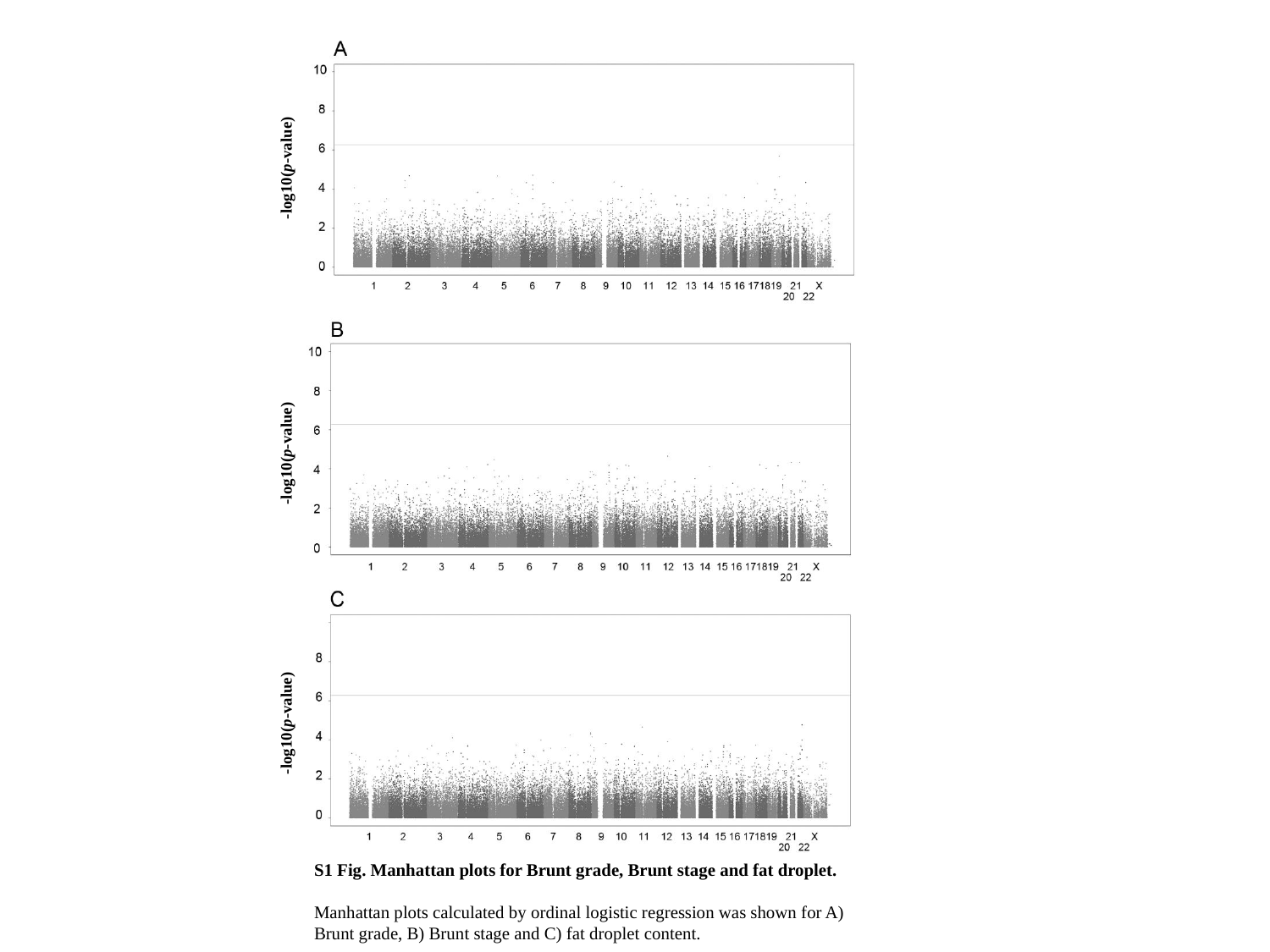

-log10(p-value)
-log10(p-value)
-log10(p-value)
S1 Fig. Manhattan plots for Brunt grade, Brunt stage and fat droplet.
Manhattan plots calculated by ordinal logistic regression was shown for A) Brunt grade, B) Brunt stage and C) fat droplet content.
