# Supplementary material for: Risk estimation model for nonalcoholic fatty liver disease in the Japanese using multiple genetic markers
Source: PLoS One. 2018 Jan 31;13(1):e0185490. doi: 10.1371/journal.pone.0185490 (PMC5791941; doi:10.1371/journal.pone.0185490)
Supplement: S2 Fig — (PPTX) [file pone.0185490.s011.pptx]

## Slide 1
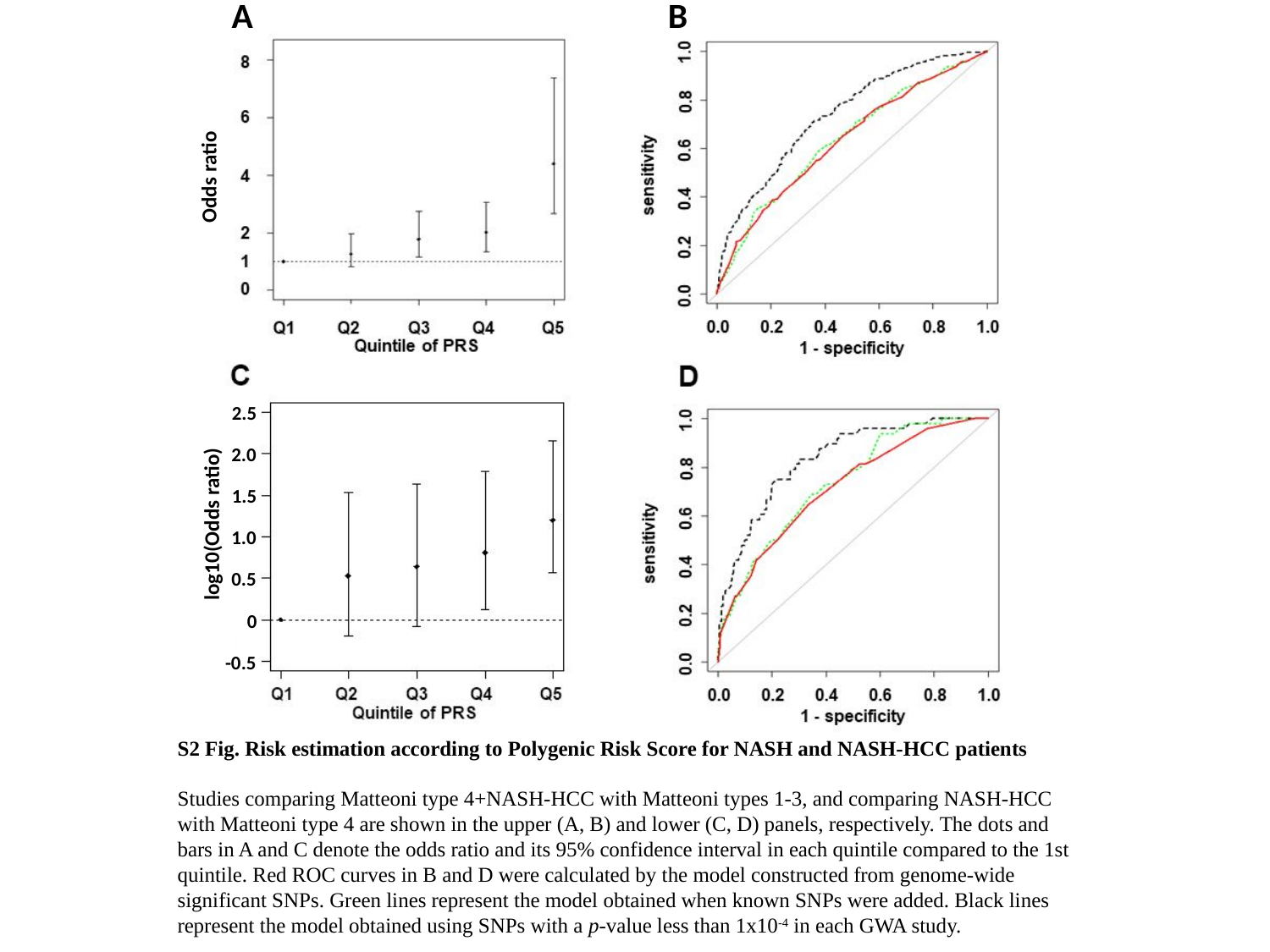

A
B
Odds ratio
2.5
2.0
1.5
log10(Odds ratio)
1.0
0.5
0
-0.5
S2 Fig. Risk estimation according to Polygenic Risk Score for NASH and NASH-HCC patients
Studies comparing Matteoni type 4+NASH-HCC with Matteoni types 1-3, and comparing NASH-HCC with Matteoni type 4 are shown in the upper (A, B) and lower (C, D) panels, respectively. The dots and bars in A and C denote the odds ratio and its 95% confidence interval in each quintile compared to the 1st quintile. Red ROC curves in B and D were calculated by the model constructed from genome-wide significant SNPs. Green lines represent the model obtained when known SNPs were added. Black lines represent the model obtained using SNPs with a p-value less than 1x10^-4^ in each GWA study.
