# Supplementary material for: Risk estimation model for nonalcoholic fatty liver disease in the Japanese using multiple genetic markers
Source: PLoS One. 2018 Jan 31;13(1):e0185490. doi: 10.1371/journal.pone.0185490 (PMC5791941; doi:10.1371/journal.pone.0185490)
Supplement: S3 Fig — (PPTX) [file pone.0185490.s012.pptx]

## Slide 1
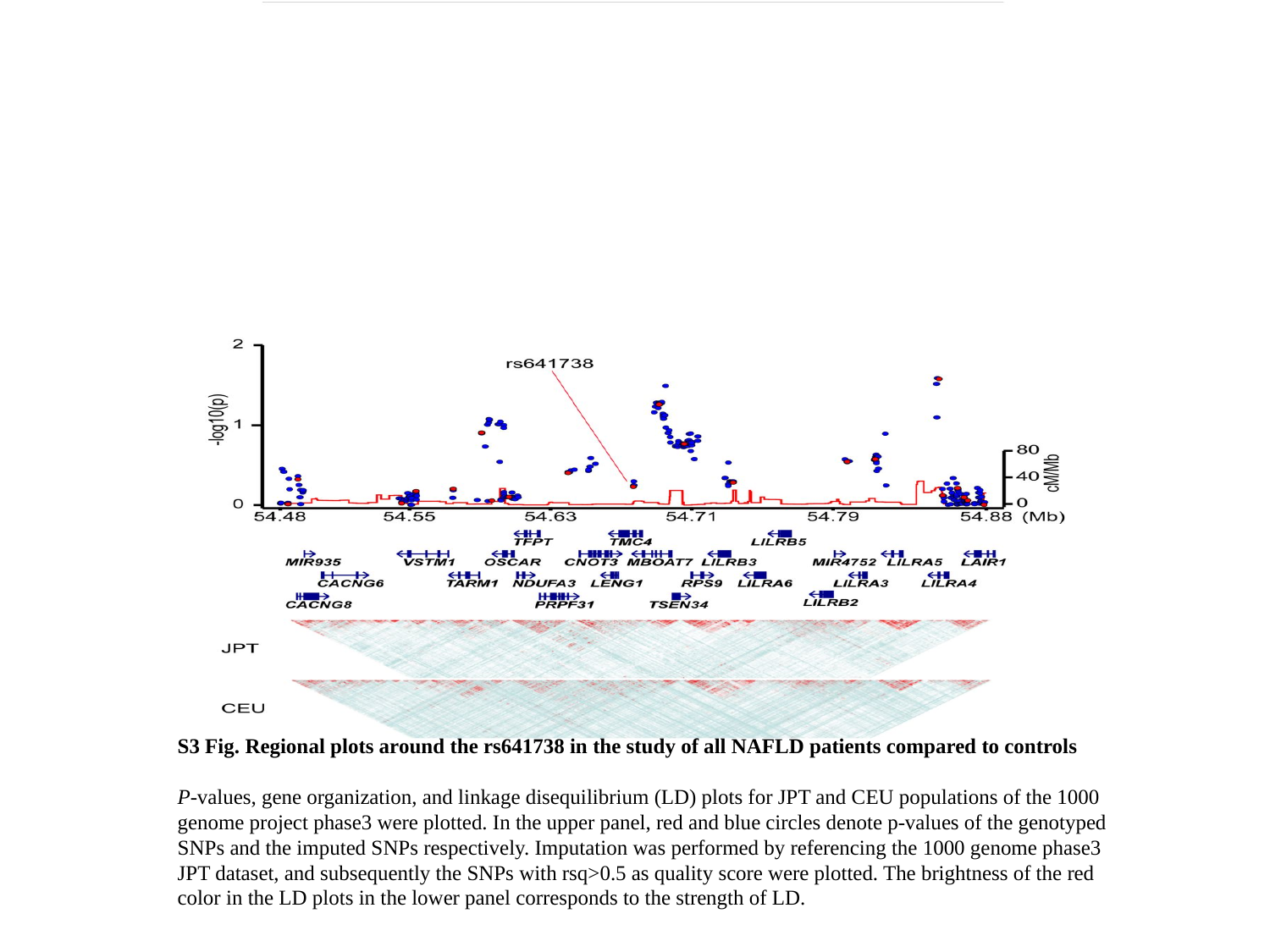

S3 Fig. Regional plots around the rs641738 in the study of all NAFLD patients compared to controls
P-values, gene organization, and linkage disequilibrium (LD) plots for JPT and CEU populations of the 1000 genome project phase3 were plotted. In the upper panel, red and blue circles denote p-values of the genotyped SNPs and the imputed SNPs respectively. Imputation was performed by referencing the 1000 genome phase3 JPT dataset, and subsequently the SNPs with rsq>0.5 as quality score were plotted. The brightness of the red color in the LD plots in the lower panel corresponds to the strength of LD.
